# The WblC/WhiB7 Transcription Factor Controls Intrinsic Resistance to Translation-Targeting Antibiotics by Altering Ribosome Composition

**DOI:** 10.1128/mBio.00625-20

**Published:** 2020-04-14

**Authors:** Ju-Hyung Lee, Ji-Sun Yoo, Yeonbum Kim, Jong-Seo Kim, Eun-Jin Lee, Jung-Hye Roe

**Affiliations:** a School of Biological Sciences, Seoul National University, Seoul, South Korea; b Institute of Microbiology, Seoul National University, Seoul, South Korea; c Center for RNA Research, Institute for Basic Science (IBS), Seoul, South Korea; d Department of Life Sciences, School of Life Sciences and Biotechnology, Korea University, Seoul, South Korea; Yonsei University

**Keywords:** *Streptomyces coelicolor*, WhiB-like protein, antibiotic resistance, ribosome-associated proteins, translation-targeting antibiotics

## Abstract

Bacteria that encounter antibiotics can efficiently change their physiology to develop resistance. This intrinsic antibiotic resistance is mediated by multiple pathways, including a regulatory system(s) that activates specific genes. In some *Streptomyces* and *Mycobacterium* spp., the WblC/WhiB7 transcription factor is required for intrinsic resistance to translation-targeting antibiotics. Wide conservation of WblC/WhiB7 within *Actinobacteria* indicates a critical role of WblC/WhiB7 in developing resistance to such antibiotics. Here, we identified 312 WblC target genes in Streptomyces coelicolor, a model antibiotic-producing bacterium, using a combined analysis of RNA sequencing and chromatin immunoprecipitation sequencing. Interestingly, WblC controls many genes involved in translation, in addition to previously identified antibiotic resistance genes. Moreover, WblC promotes translation rate during antibiotic stress by altering the ribosome-associated protein composition. Our genome-wide analyses highlight a previously unappreciated antibiotic resistance mechanism that modifies ribosome composition and maintains the translation rate in the presence of sub-MIC levels of antibiotics.

## Abstract (precis)

The emergence of antibiotic-resistant bacteria is one of the top threats in human health. Therefore, we need to understand how bacteria acquire resistance to antibiotics and continue growth even in the presence of antibiotics. Streptomyces coelicolor, an antibiotic-producing soil bacterium, intrinsically develops resistance to translation-targeting antibiotics. Intrinsic resistance is controlled by the WblC/WhiB7 transcription factor that is highly conserved within *Actinobacteria*, including Mycobacterium tuberculosis. Here, identification of the WblC/WhiB7 regulon revealed that WblC/WhiB7 controls ribosome maintenance genes and promotes translation in the presence of antibiotics by altering the composition of ribosome-associated proteins. Also, the WblC-mediated ribosomal alteration is indeed required for resistance to translation-targeting antibiotics. This suggests that inactivation of the WblC/WhiB7 regulon could be a potential target to treat antibiotic-resistant mycobacteria.

## INTRODUCTION

Bacteria that produce antibiotics or antimicrobials to limit the growth of other microbial species often develop antibiotic resistance to protect themselves from antibiotics. This self-defense mechanism is called intrinsic resistance (1, 2). The intrinsic antibiotic resistance mechanism includes a physical barrier(s) decreasing permeability of drugs, an efflux pump decreasing the cytoplasmic concentration of antibiotics, an enzyme(s) inactivating the action of antibiotics, or a physiological adaptation(s) resolving cellular stresses mediated by antibiotics (1, 3). Such intrinsic antibiotic resistance relies on a regulatory protein(s) that activates expression of a specific set of genes in response to antibiotics. *Actinomycetes*, specifically the genus *Streptomyces* producing three-fourths of all known antibiotics (4, 5) and pathogenic mycobacteria, including Mycobacterium tuberculosis, are intrinsically resistant to many antibiotics (3, 6). The retention of such intrinsic resistance in both organisms depends on the WblC/WhiB7 transcription factor, which controls expression of several genes involved in antibiotic resistance (7–9).

WblC is a WhiB-like transcriptional regulator in *Streptomyces* that is widely conserved among many actinomycetes (10, 11). WhiB7, its ortholog in mycobacteria, has been extensively studied for its role as a transcriptional activator of antibiotic resistance (8, 9, 12). The mycobacterial WhiB7 protein has a Fe-S cluster binding domain with four conserved cysteine residues, a binding domain for the SigA housekeeping sigma factor, and an AT-hook DNA-binding region with positively charged residues, all of which are required for its function (8, 13). Strains lacking WhiB7/WblC are highly susceptible to a broad spectrum of translation-targeting antibiotics (8, 9, 12, 14, 15), and transcription of the *whiB7* or *wblC* gene itself is induced up to about 500-fold upon treatment with the same translation-targeting antibiotics (16, 17), suggesting that WhiB7/WblC is required for resistance to translation-targeting antibiotics. Interestingly, WhiB7 inducers also include antibiotics inhibiting DNA replication and metabolism, and physiological stresses inhibiting bacterial growth such as iron starvation, heat shock, and stationary phase (16, 18) that might indirectly affect ribosome availability.

Previous studies to search the WhiB7 regulon identified numerous WhiB7 target genes in mycobacteria (9, 12). Morris et al. reported 12 genes as targets using the wild-type, *whiB7* deletion mutant, or *whiB7*-complemented strains from M. tuberculosis upon ribosome-targeting antibiotic treatment (9). However, the RNA profiles relied on microarray analysis with limited resolution. Hurst-Hess et al. reported about 100 differentially expressed genes (DEGs) using RNA sequencing of the wild-type, *whiB7* deletion, and *whiB7*-complemented strains from Mycobacterium abscessus and Mycobacterium smegmatis without antibiotic treatment (12). Given that *whiB7* expression itself is highly induced upon ribosome-targeting antibiotic treatment (7, 9, 12, 17), it is more reasonable to search for WhiB7/WblC target genes in a condition that bacteria are treated with antibiotics. In addition, previous studies might include a list of genes that are indirectly controlled by WhiB7/WblC. Collectively, further extensive analyses are needed to unveil the direct target genes and understand the role of WhiB7/WblC in *Actinobacteria*. Here, we defined direct WblC targets in Streptomyces coelicolor by comparing the transcriptome sequencing (RNA-seq) and chromatin immunoprecipitation-sequencing (ChIP-seq) profiling of the wild-type and *wblC* mutant strains in the presence of antibiotics. We identified 312 genes as a bona fide WblC regulon that is directly controlled by WblC. We also examined how WblC contributes to activating the majority of these genes on promoters recognized by HrdB, the housekeeping sigma factor and how WblC regulon members contribute to translation-inhibiting antibiotic resistance, especially via altering ribosome composition.

## RESULTS

### WblC remodels global transcription in response to antibiotic stress.

**FIG 1:**
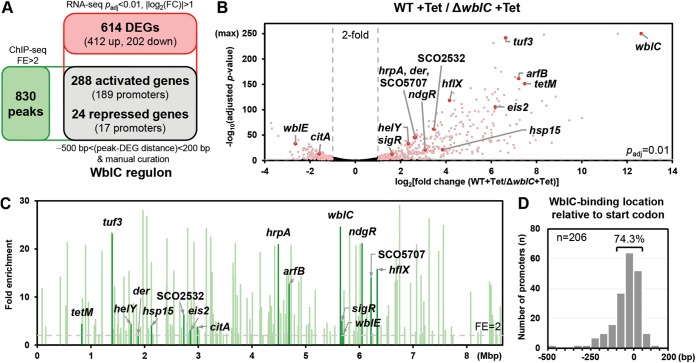
Genome-wide identification of the WblC regulon. (A) Defining the WblC regulon. A total of 614 differentially expressed genes (DEGs) (412 genes upregulated and 202 genes downregulated by *wblC* induction) were identified by RNA-seq, and 830 WblC binding peaks were identified by ChIP-seq. A total of 312 genes (288 upregulated and 24 downregulated) at the intersection of two data sets were defined as the WblC regulon. (B) Volcano plot comparing RNA-seq profiles of wild-type (WT) and Δ*wblC* cells treated with 2 μg/ml tetracycline (Tet) for 30 min. DEGs (pink) are defined by the adjusted *P* value (*p*_adj_ < 0.01, horizontal gray dashed line) and fold change cutoff (|log_2_ fold change | > 1, vertical gray dashed lines). Selected WblC regulon genes are labeled and dotted in red. (C) Genome-wide ChIP peak distribution of the WblC regulon. Genes above the fold enrichment cutoff (FE > 2; gray dashed line) are displayed. Peaks of selected WblC regulon genes are labeled and colored in dark green lines. (D) Distribution of WblC binding summits among the WblC regulon. WblC binding summits are indicated as the location relative to the start codon of the first gene in each operon. The total number of peaks (n) is indicated.

Given that WblC is required for resistance to ribosome-targeting antibiotics (8, 9, 12, 19), we expected that WblC might have an impact on genome-wide S. coelicolor gene expression in response to ribosome-targeting antibiotics. To identify WblC targets in S. coelicolor, we performed RNA sequencing of the wild-type and Δ*wblC* strains treated with or without tetracycline. We obtained biologically independent duplicates for each condition. The transcription profiles of the duplicates were highly correlated with each other for all four conditions (*R*^2^ > 0.9), showing that there was little batch-to-batch variation (see Fig. S1A to S1D in the supplemental material). Principal-component analysis of the samples revealed that large differences were observed between the transcriptome profiles of the tetracycline-treated and untreated wild-type strain, whereas the profiles of untreated samples from the wild-type and Δ*wblC* strains were clustered together (Fig. S1E). When treated with 2 μg/ml tetracycline (0.2× MIC) for 30 min, a total of 2,908 genes (1,417 genes upregulated and 1,491 genes downregulated) were affected by tetracycline treatment in the wild type (>2-fold, adjusted *P* value <0.01) (GEO accession number GSE136168). Among these genes, transcript levels of 614 genes out of 7,853 open reading frames (ORFs) (excluding pseudogenes) were significantly different (adjusted *P* value of <0.01) by >2-fold between the wild-type and Δ*wblC* strains (Fig. 1A and B). Because WblC affects about 7.8% of the genome, it indicates that WblC extensively alters the S. coelicolor transcriptome when cells are treated with tetracycline. Among 614 genes, WblC upregulated 412 genes and downregulated 202 genes in the presence of tetracycline (Fig. 1A). In contrast, only 67 genes were significantly different (adjusted *P* value of <0.01) by >2-fold when we compared untreated samples of wild-type and Δ*wblC* strains (Fig. S1F). Moreover, the fold changes of the untreated samples between the wild-type and Δ*wblC* strains were distributed within a smaller range (from a 7-fold increase to 9-fold decrease) compared to those of tetracycline-treated samples, which ranged from a 3,000-fold increase to 13-fold decrease (Fig. 1B and Fig. S1F). Consistent with a previous finding that WblC proteins are not detected by immunoblotting unless WblC production is induced by translation-inhibiting antibiotic treatment (19), these data suggest that steady-state WblC levels in unstressed conditions exhibit only a marginal effect on the S. coelicolor transcriptome.

10.1128/mBio.00625-20.2 FIG S1 RNA-seq sample statistics. (A to D) Scatter plots of log_10_ normalized count between biological duplicates of untreated wild-type (A), tetracycline-treated wild-type (B), untreated Δ*wblC* (C), and tetracycline-treated Δ*wblC* (D) strains. Genes excluding pseudogenes and rRNA genes were plotted. Linear regression analysis between biological duplicates showed the coefficient of determination (*R*^2^) values of 0.97 (A), 0.95 (B), 0.94 (C), and 0.95 (D), suggesting that RNA-seq results are highly reproducible. (E) Principal component analysis of the eight RNA-seq samples. The fractions of variance explained by each component are denoted on each axis. It resulted in three distinct clusters among untreated strains, tetracycline-treated wild-type, and tetracycline-treated Δ*wblC* strains. (F) Fewer genes are affected by *wblC* deletion in the absence of tetracycline. Volcano plot comparing RNA-seq profiles of wild-type and Δ*wblC* strains in the absence of tetracycline. DEGs (pink) are defined by the adjusted *P* value (*p*_adj_ < 0.01, horizontal gray dashed line) and fold change cutoff (|log_2_ fold change| > 1, vertical gray dashed lines). The *wblC* gene exhibiting decreased expression in the Δ*wblC* strain and the SCO5191 gene exhibiting increased expression in the Δ*wblC* strain are indicated. (G) Distribution of WblC binding summits among the WblC-repressed genes. WblC binding summits are indicated as the location relative to the start codon of the first gene in each operon. The total number of promoters (n) is indicated. (H) WblC’s binding to selected promoters is confirmed by ChIP-qPCR. The ChIP-qPCR assay was performed in wild-type and Δ*wblC* strains treated with 2 μg/ml tetracycline (Tet) for 1 h. Relative percent input represents percent input in wild-type strain/percent input in Δ*wblC* strain in the presence of tetracycline. Shown are means ± SE from three independent experiments (*P* < 0.05). Download FIG S1, PDF file, 0.6 MB. Copyright © 2020 Lee et al. 2020 Lee et al. This content is distributed under the terms of the Creative Commons Attribution 4.0 International license.

### A large number of genes are directly controlled by WblC.

**FIG 2:**
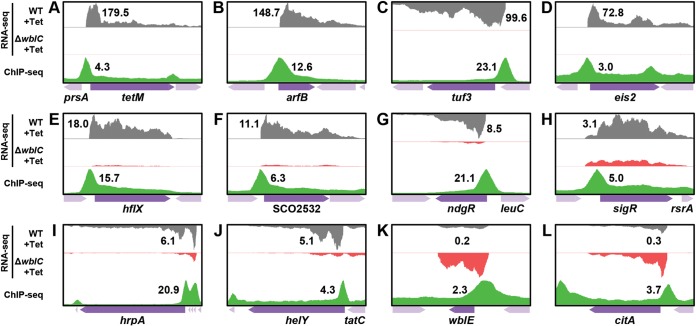
Examples of selected WblC regulon. (A) *tetM*, (B) *arfB*, (C) *tuf3*, (D) *eis2*, (E) *hflX*, (F) *SCO2532*, (G) *ndgR*, (H) *sigR*, (I) *hrpA*, and (J) *helY* are WblC-activated genes encoding a tetracycline resistance protein, a peptidyl-tRNA hydrolase, an elongation factor Tu3, an aminoglycoside resistance protein, a ribosome-associated GTPase, a PhoH-like protein, a transcription regulator involved in leucine biosynthesis, a redox-sensing extracytoplasmic function (ECF) sigma factor, an ATP-dependent helicase, and an RNA helicase, respectively. (K) *wblE* and (L) *citA* are WblC-repressed genes encoding a WhiB-paralogous protein and CitA citrate synthase, respectively. Visualized sequence reads of the WblC regulon genes and surrounding regions (± 600 bp) are presented. The heights of RNA sequencing reads of each panel are normalized to the peak showing the highest expression within the panel. The highest values of fold change in RNA-seq and fold enrichment in ChIP-sequencing are shown in each panel. Dark purple arrows indicate WblC regulon genes, and pale purple arrows indicate their neighboring genes.

The 614 differentially expressed genes (DEGs) identified by RNA sequencing were likely to encompass both direct and indirect WblC targets. To find direct WblC targets, we performed ChIP-seq in wild-type cells treated with tetracycline and identified WblC binding sites across the genome. We defined 830 peaks as WblC binding sites that were enriched more than twofold compared to control input DNA (Fig. 1A). We combined ChIP-seq data with RNA-seq data to select direct WblC targets. We first examined the sequence from −500 to +200 bp relative to the start codon of 614 DEGs identified from RNA-seq data to assess whether the selected DEGs contained WblC binding peaks. Then, the selected DEGs were further curated to remove false-positive peaks, including instances where the binding peak belonged to a divergently transcribed gene(s). Using this procedure, we identified 206 promoters containing WblC binding sites (Fig. 1C) and assigned 312 DEGs to the WblC regulon (Fig. 1A; see also Table S2 in the supplemental material). Of the WblC binding peaks, 74.3% were located from −100 to +50 bp relative to the start codon of the first gene in each operon (Fig. 1D). Among 312 genes, the RNA levels of 288 genes were higher in the tetracycline-treated samples from the wild-type strain than those from the Δ*wblC* strain (represented in Fig. 2A to J), indicating that WblC functions as a transcriptional activator of those genes. Interestingly, RNA levels of 24 genes were lower in the tetracycline-treated samples from the wild-type strain than those from the Δ*wblC* strain (represented in Fig. 2K and L), suggesting that WblC could also act as a transcriptional repressor of those genes. WblC binding sites of repressed genes were mostly located upstream of the translation start codon, lying between −500 and 0 bp relative to the translation start codon (Fig. S1G). WblC binding of the selected promoters was further confirmed by ChIP-qPCR (Fig. S1H).

### WblC regulon products could antagonize antibiotic stress by reducing the concentration of effective antibiotics, enhancing translation, resolving oxidative stress, and adjusting metabolic pathways.

**FIG 3:**
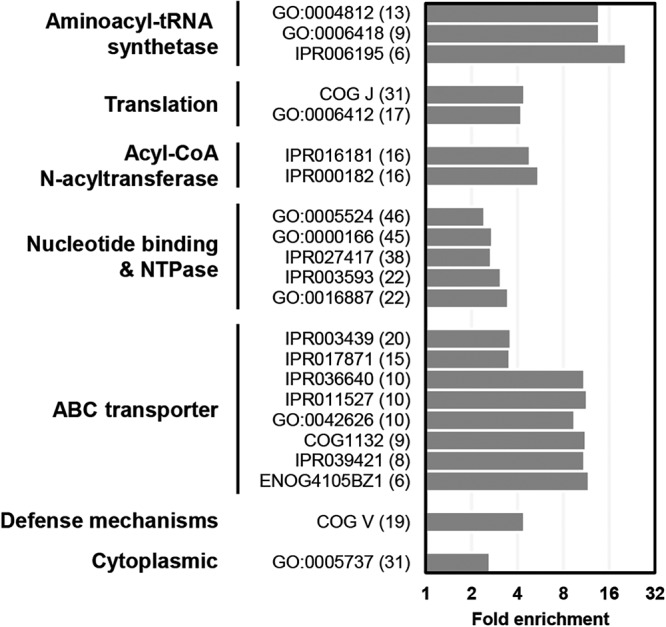
Functional classification of WblC-activated genes. Gene Ontology (GO) terms, InterPro (IPR) entries, and EggNOGs (ENOG and COG) significantly enriched (adjusted *P* value < 0.05) in the WblC-activated regulon relative to the chromosomal protein-coding genome are presented. The number of WblC-activated regulon genes in each functional class is indicated in parentheses.

We then tried functional annotations of the WblC regulon using Gene Ontology (GO) terms, InterPro entries, and EggNOGs. Analyses of the WblC-activated genes revealed a significant enrichment in several functions, including tRNA aminoacylation, translation, acyl-coenzyme A (acyl-CoA)-dependent N-acyltransferase, nucleoside triphosphate hydrolase (NTPase) with nucleotide binding, and ABC transporter (Fig. 3). After performing hierarchical clustering, we further selected 179 WblC-activated genes and classified them into eight major categories with some subcategories (Table 1). The remaining 109 genes were uncategorized mostly due to insufficient annotations.

**TABLE 1:** Functional classification of WblC-activated genes based on annotated functions

Category (no. of genes)^*a*^	Representative gene(s)
1. Aminoacyl-tRNA synthesis and editing enzymes (16)	*thrS2*, *lysS*, *alaS2*, *trpS*
2. P-loop NTPases (35)	
2A. ABC transporters with transmembrane domains (10)	SCO1147, SCO5451
2B. ABC transporter ATP-binding cassette proteins (10)	SCO6512, SCO3824
2C. Other ATPases (9)	SCO2532, *hrpA*, *helY*
2D. GTPases (6)	*hflX*, *der*, *tetM*, *tuf3*
3. Transferases (32)	SCO4264, SCO7710
3A. Gcn5-related *N*-acetyltransferases (16)	*eis*, *eis2*
3B. Methyltransferases (7)	*lrm*
4. Integral membrane proteins (39)	SCO2896, SCO1362
4A. Major facilitator superfamily (11)	*cmlR2*, *pep*
5. Oxidoreductases (16)	*asd1*, *pntA*
6. Hydrolases (16)	*arfB*
7. Transcription regulators (19)	*wblC*, *sigR*, *ndgR*
8. tRNAs (6)	
Others (109)	*hsp15*, SCO5707, *vgb*, *smpB*

a Genes were grouped by similar functional categories. The number of genes in each group is indicated in parentheses.

On the basis of the functional categorizations, we reasoned how WblC-activated genes are involved in resistance to translation-targeting antibiotics. ABC transporter proteins, major facilitator superfamily proteins such as CmlR2 (20) or Pep (21), and other membrane proteins could decrease the intracellular antibiotic concentration by exporting antibiotics. Eis and Eis2 aminoglycoside acetyltransferases (22), virginiamycin B lyase (Vgb) (23), and possibly other transferases could modify antibiotics to inactivate their functions. Interestingly, WblC also activates genes encoding aminoacyl-tRNA synthetases, tRNA processing enzymes, several amino acid biosynthetic enzymes, and tRNAs (Table 1 and Table S2). It can be hypothesized that these enzymes supply the needed aminoacyl-tRNAs even in the tetracycline-treated conditions to maintain translation. Moreover, WblC increases mRNAs of genes encoding Erm(O)-type rRNA methyltransferases (encoded by SCO6089) homologous to Streptomyces lividans Lrm (24), TetM ribosome-associated GTPase (25), several Gcn5-related *N*-acetyltransferases, and other proteins like SmpB (26) (Table 1). The functions of those genes are predicted to modify, modulate, or protect translational machinery against antibiotics, supporting the idea that WblC is involved in resuming translation during translational stress mediated by antibiotics. Additionally, it suggests that 19 transcriptional regulators, including WblC itself (11), SigR redox- and antibiotic-responsive sigma factor (27), and NdgR involved in leucine biosynthesis (28), play roles in resolving oxidative stress and adjusting metabolic pathways generated by translation-targeting antibiotics. Cumulatively, these findings imply that WblC-activated genes are involved in a protective response to translation-interrupting antibiotics to maintain viability, which could be an intrinsic resistance mechanism to translation-targeting antibiotics. In contrast to WblC-activated genes, WblC-repressed genes were not significantly enriched in any of the GO terms, InterPro entries, or EggNOGs classifications.

### WblC controls 22 noncoding RNAs.

We noticed that 22 noncoding RNAs, including six tRNA genes are directly regulated by WblC (Table S3). Four small noncoding RNAs are located independently in the intergenic regions (Fig. S2A and S2B and Table S3), suggesting that they could function as *trans*-acting riboregulators. Twelve noncoding RNAs are mapped to the antisense strand of the neighboring gene(s), which could be designated as *cis*-acting antisense RNAs that could downregulate expression of the overlapping genes (Fig. S2C and S2D and Table S3). Interestingly, even among the identified WblC regulon, 104 mRNAs have 5′ or 3′ long untranslated regions (UTRs) that overlap a gene(s) in the opposite strand and thus could also function as antisense RNAs of the neighboring gene(s) (29, 30) (Fig. S2E and S2F and Table S3). In conclusion, these results indicate that WblC not only controls mRNA transcriptome but also controls expression of many noncoding RNAs and UTRs, possibly adding another layer of gene regulation during antibiotic stress.

10.1128/mBio.00625-20.3 FIG S2 WblC controls noncoding RNAs and antisense RNAs. (A and B) Examples of noncoding RNAs located in the intergenic region between SCO4122 and *rrnA* (A) or downstream of SCO5111 (B). (C and D) Examples of antisense RNAs with independent promoters overlapping with SCO3960 (C) or SCO4388 (D). (E and F) Examples of antisense RNAs originated from UTRs of the neighboring SCO0909 (E) and SCO1988 (F) genes. Download FIG S2, PDF file, 0.4 MB. Copyright © 2020 Lee et al. 2020 Lee et al. This content is distributed under the terms of the Creative Commons Attribution 4.0 International license.

### Conserved sequence elements in the WblC-activated promoters suggest a mechanism of promoter recognition by WblC-HrdB interaction.

**FIG 4:**
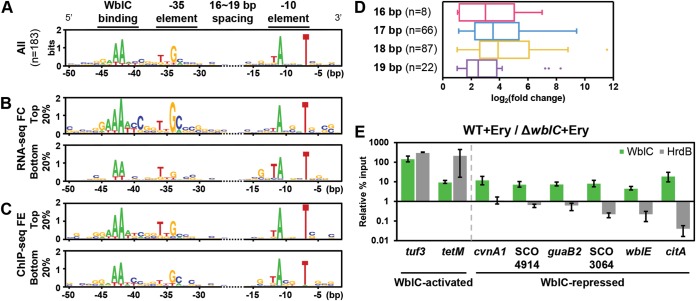
The conserved binding motif of WblC-activated genes. (A to C) Conserved promoter sequences of the WblC-activated genes. (A) Conserved WblC-binding motif (GAAAWY), −35 motif (TKGMNN), and −10 motif (TANNNT) were identified among 183 WblC-activated promoters and visualized by a sequence logo. The distances between the −35 and −10 motifs of the WblC-activated promoters range from 16 to 19 bp. Degenerate base codes in WblC-activated promoter consensus sequence are as follows: W (A or T), Y (C or T), K (T or G), M (C or A), and N (any nucleotide). (B) Conserved sequence motifs among the top 20% or bottom 20% WblC-activated genes in RNA-seq profiles. FC, fold change. (C) Conserved sequence motifs among top 20% or bottom 20% WblC-activated genes in ChIP-seq profiles. The values and scales of the *x* axis and *y* axis in panels B and C are the same as in panel A. FE, fold enrichment. (D) Distribution of RNA fold changes of WblC-activated genes relative to the spacing between the −10 and −35 motifs. In each spacing groups, RNA fold changes (log_2_ fold change) of the first gene in each transcriptional unit (TU) are presented as a box-and-whisker plot. The number of promoters in each group are denoted. (E) HrdB is enriched in the WblC-activated promoters but not in the WblC-repressed promoters. The ChIP-qPCR assay was performed in wild-type and Δ*wblC* strains treated with 1 μg/ml erythromycin (Ery) for 1 h. Relative percent input represents the percent input in wild-type cells/percent input in Δ*wblC* cells in the presence of erythromycin. Please note that the relative percent input of HrdB in the promoter regions of SCO3064, *wblE*, and *citA* genes is less than 1 because HrdB was enriched more in the Δ*wblC* mutant than in the wild type. Values are means ± standard errors (SE) (error bars) from three biologically independent experiments.

In mycobacteria, WhiB7 binds to a conserved AT-rich sequence adjacent to −35 promoter elements (16), and WhiB7’s binding to the AT-rich motif enables a cooperative binding between WhiB7, SigA sigma factor, and the target promoters (13). Similarly, we examined the promoter regions of WblC targets in S. coelicolor to look for a conserved motif. MEME analysis of 189 WblC-activated promoters revealed that WblC motifs are located 3 bp upstream of the −35 element, and the spacing between the −35 and −10 elements is between 16 and 19 bp (Fig. 4A). The optimal spacer appears to be 17 or 18 bp because WblC promoters with the 17- or 18-bp spacer are most abundant and showed a higher fold change in RNA-seq than those with the 16- or 19-bp spacer (Fig. 4D). The −10 elements of WblC-activated promoters are more conserved, similar to the genome-wide consensus (TANNNT) (31), whereas the −35 elements are less conserved. Most of the transcription start sites (TSSs) are located 7 or 8 bp downstream of the −10 elements, which is consistent with previously reported transcriptional profiling in S. coelicolor (31) (Fig. S3).

10.1128/mBio.00625-20.4 FIG S3 Positions of −10 motifs relative to TSS. The promoters (*n* = 105) with identified −10 motifs were grouped according to the position relative to TSS. When −10 motif and reported TSS are >50 bp apart, those −10 motifs were excluded from the analysis. Download FIG S3, PDF file, 0.2 MB. Copyright © 2020 Lee et al. 2020 Lee et al. This content is distributed under the terms of the Creative Commons Attribution 4.0 International license.

There appears to be a clear correlation between the WblC-activated promoter sequences and mRNA fold changes. When we compared the promoter sequences of genes showing the top 20% and bottom 20% fold changes in RNA-seq, we observed that the top 20% of the WblC-activated promoter sequences exhibited a higher enrichment of A nucleotides in the WblC binding sites than those in the bottom 20% (Fig. 4B). High expression levels are also likely to be linked to other features, including appearance of pyrimidines following stretches of A nucleotides within the WblC binding sites, a higher enrichment of G at the third position of the −35 element, and a lower enrichment of T at the first position of the −10 element (Fig. 4B). A similar sequence correlation was observed in the promoter sequences showing the top 20% fold enrichment in ChIP-seq and top 20% in RNA-seq (Fig. 4B and C).

In contrast, there is no distinctive feature in the WblC-repressed promoters, suggesting that the binding mode of the WblC-repressed genes might differ from those of the WblC-activated genes. Given that, in the WblC-activated promoters, WblC binding sites are adjacent to the −35 elements of the promoter sequences to facilitate a cooperative binding of WblC and the housekeeping sigma factor HrdB (SigA for M. tuberculosis) to the promoters (Fig. 4A to C), we tested whether such cooccurrences of the WblC binding sites and HrdB-binding promoter elements were detected in the WblC-repressed promoters by ChIP-quantitative PCR (qPCR) assay. As expected, WblC-activated promoters were enriched in both the WblC-immunoprecipitated and HrdB-immunoprecipitated DNA samples (Fig. 4E, *tuf3* and *tetM* promoters). However, HrdB seems not to colocalize with WblC in the WblC-repressed promoters because WblC-repressed promoters were enriched only in the WblC-immunoprecipitated DNA samples, not in the HrdB-immunoprecipitated DNAs (Fig. 4E, *cvnA1*, *SCO4914*, *guaB2*, *SCO3064*, *wblE*, and *citA* promoters).

### *wblC* is required to maintain translation and growth rate against subinhibitory concentrations of antibiotics.

**FIG 5:**
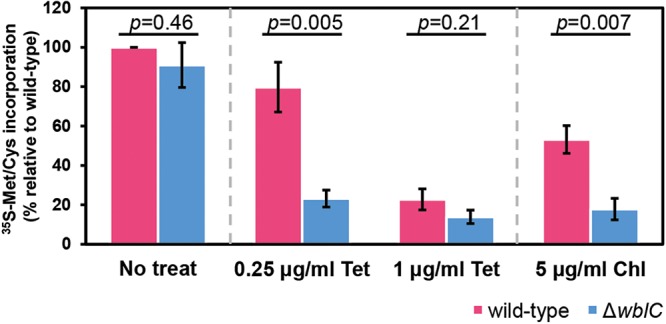
*wblC* is required for maintaining the translation rate against sub-MIC levels of translation-inhibiting antibiotics. The translation rate of the wild-type or Δ*wblC* strain was measured using the incorporation rate of ^35^S-labeled methionine/cysteine from cells treated with tetracycline (Tet) or chloramphenicol (Chl) at the indicated concentrations. Cells were treated with tetracycline and chloramphenicol for 1 h and then normalized to measure [^35^S]Met/Cys incorporation rate as described in Materials and Methods. The incorporation rate of the untreated wild-type strain (No treat) was set at 100% and compared to others as relative ratios. The values shown are means ± SE from four independent experiments. *P* values from two-tailed Student’s *t* test are indicated. See Fig. S4C to F for source data.

Given that *wblC* expression itself is highly induced upon translation-inhibiting antibiotic treatment (19) and a significant portion of the identified *wblC* regulon is involved in translation, we wondered whether WblC-controlled genes could actually promote translation rate in response to antibiotic stress. We measured the incorporation rate of ^35^S-radiolabeled methionine and cysteine in the wild-type and Δ*wblC* cells after treating them with tetracycline or chloramphenicol for 1 h. When treated with tetracycline at a subinhibitory concentration (0.25 μg/ml; Fig. S4A), wild-type cells still maintained 80% of [^35^S]Met/Cys incorporation rate compared to untreated cells, whereas Δ*wblC* cells had a significantly decreased incorporation rate that was approximately fourfold lower than that of the untreated control (Fig. 5). Likewise, chloramphenicol, another translation-targeting antibiotic, exhibited a similar decrease in translation when treated with Δ*wblC* cells at a subinhibitory concentration (5 μg/ml [Fig. 5 and Fig. S4B]). Considering that global translation efficiency is one of the major limiting factors determining growth rate (32, 33), this suggests that WblC-controlled genes are responsible for maintaining translation and growth rate during translational stress mediated by antibiotics. Control experiments demonstrated the following: the incorporation rates in wild-type cells and Δ*wblC* cells were similar to each other and remained high in the untreated condition. However, the incorporation rates in both wild-type cells and Δ*wblC* cells decreased severely when treated with tetracycline at a lethal concentration (1 μg/ml [Fig. 5 and Fig. S4]).

10.1128/mBio.00625-20.5 FIG S4 WblC affects *Streptomyces* growth and translation rate at sub-MIC levels of antibiotics. (A and B) Growth curves of wild-type and Δ*wblC* strains in YEME liquid medium untreated or treated with tetracycline (A) or chloramphenicol (B) at the indicated concentrations. Bacteria were grown at 37°C for 15.5 h with shaking after spore inoculation. After antibiotics were treated at 15.5 h (indicated by arrows), growth was monitored for an additional 6 h. Shown are means ± SE from three independent experiments. (C to F) Autoradiographs of four independent experiments of *in vivo* [^35^S]Met/Cys pulse-chase labeling, related to Fig. 5. Signal intensities of the untreated wild-type strain were set at 1 and compared to others as relative ratios. Download FIG S4, PDF file, 0.5 MB. Copyright © 2020 Lee et al. 2020 Lee et al. This content is distributed under the terms of the Creative Commons Attribution 4.0 International license.

### WblC alters the composition of ribosome-associated proteins upon antibiotic stress.

**FIG 6:**
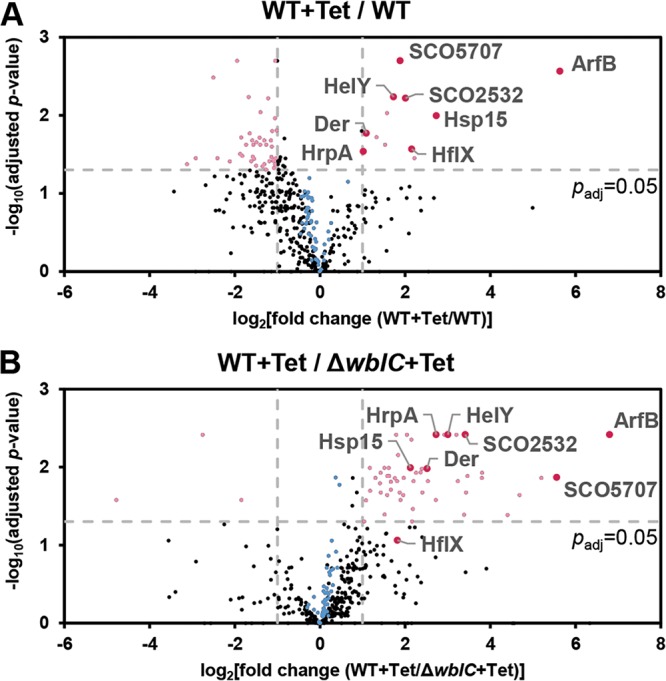
*wblC* is required for altering the composition of ribosome-associated proteins during tetracycline treatment. (A) Volcano plot analysis of ribosome-associated proteins in the wild-type strain not treated or treated with tetracycline. (B) Volcano plot analysis of ribosome-associated proteins in wild-type and Δ*wblC* strains treated with tetracycline. Mass spectrometry data of each sample with an adjusted *P* value of <0.05 and |log_2_ fold change| > 1 are dotted in magenta. WblC-dependent proteins among the identified ribosome-associated proteins are colored in dark magenta, labeled, and presented in Table 2. Blue dots represent ribosomal proteins.

On the basis of the facts that WblC controls many genes involved in translation and that WblC promotes translation rate upon antibiotic stress, we suspected that WblC might actually alter the composition of the ribosome during antibiotic stress. To test this idea, we prepared 70S ribosome fractions from wild-type cells untreated or treated with tetracycline and Δ*wblC* mutant cells treated with tetracycline and compared their protein compositions by mass spectrometric quantification. In ribosomal fractions from wild-type cells, we found that 12 ribosome-associated proteins were significantly enriched in the tetracycline-treated sample compared to the untreated control (Fig. 6A). The alteration in the ribosomal composition depends on WblC because, even in the tetracycline-treated condition, ribosomal fractions prepared from the Δ*wblC* mutant did not accumulate the above-mentioned ribosome-associated proteins compared to the levels in tetracycline-treated wild-type strain (Fig. 6B). Notably, in tetracycline-treated samples, we found 54 proteins significantly enriched in the wild-type ribosome fractions but not in the Δ*wblC* ribosome fractions (Fig. 6B).

We were particularly interested in 10 ribosome-associated proteins that were enriched in a tetracycline- and WblC-dependent manner (Table 2). Among those 10 proteins, eight proteins are encoded by WblC-activated genes, supporting that WblC modulates the ribosome composition during antibiotic stress. Among them, YbeZ (SCO2532) and HrpA were identified by genome-wide studies to interact with multiple ribosomal proteins in Escherichia coli (34–36). HflX is a GTPase that is also known to be associated with the 50S ribosomal subunit (37). ArfB and Hsp15 are ribosome-associated factors that could restore a stalled ribosome or nonfunctional ribosome subunit (38–40) during antibiotic stress. Der (EngA) is an essential ribosome-binding GTPase (41, 42) that could be involved in ribosome biogenesis during antibiotic stress because EngA depletion showed a pleiotropic effect caused by a defect in ribosome biogenesis (43, 44). SCO5707 is a small conserved hypothetical protein that shares homology with *rimP* (encoding 30S ribosomal subunit maturation factor), *infB* (encoding translation initiation factor IF-2), *rbfA*, and *truB*, suggesting a possible role in protein synthesis. Although transcription of the *hpf* gene that encodes a ribosome hibernation promoting factor (HPF) involved in 70S ribosome dimerization (45, 46) is not *wblC* dependent (Table 2), enrichment of HPF in the tetracycline-treated ribosome fractions suggests that translation-inactive ribosome dimers could be formed during antibiotic stress.

**TABLE 2:** Ribosome-associated proteins that are altered in wild-type or Δ*wblC* strains during tetracycline treatment

Protein	Gene ID^*a*^	Description^*b*^	LC-MS/MS fold change^*c*^		RNA-seq fold change for WT+Tet/Δ*wblC*+Tet	WblC regulon
			WT+Tet/WT	WT+Tet/Δ*wblC*+Tet		
HrpA	SCO4092	ATP-dependent RNA helicase	2.0	6.6	6.1	+
SCO2532	SCO2532	PhoH-like protein, ortholog of E. coli YbeZ*	4.0	10.6	11.1	+
HflX	SCO5796	50S ribosomal subunit-associated GTPase	4.5	3.5	18.0	+
ArfB	SCO4278	Alternative ribosome rescue factor B*	49.7	111.4	148.7	+
HelY	SCO1631	ATP-dependent RNA helicase	3.3	8.0	5.1	+
Hsp15 (HslR)	SCO1991	Ribosome-associated heat shock protein	6.6	4.4	14.5	+
Der (EngA)	SCO1758	Ribosome-associated GTPase	2.1	5.7	6.3	+
SCO5707	SCO5707	Uncharacterized protein, with DUF503	3.7	47.2	6.2	+

HPF	SCO3009	Ribosome hibernation promoting factor	3.0	7.6	1.0	
ScoF4	SCO4295	Cold shock protein	2.5	10.8	0.9	

a ID, identifier.

b Genes annotated based on homology in the phylogenetic tree are indicated by an asterisk.

c Fold change for the wild-type (WT) strain or Δ*wblC* strain grown with tetracycline (Tet).

### WblC-dependent ribosomal alteration is involved in resistance to translation-targeting antibiotics.

**FIG 7:**
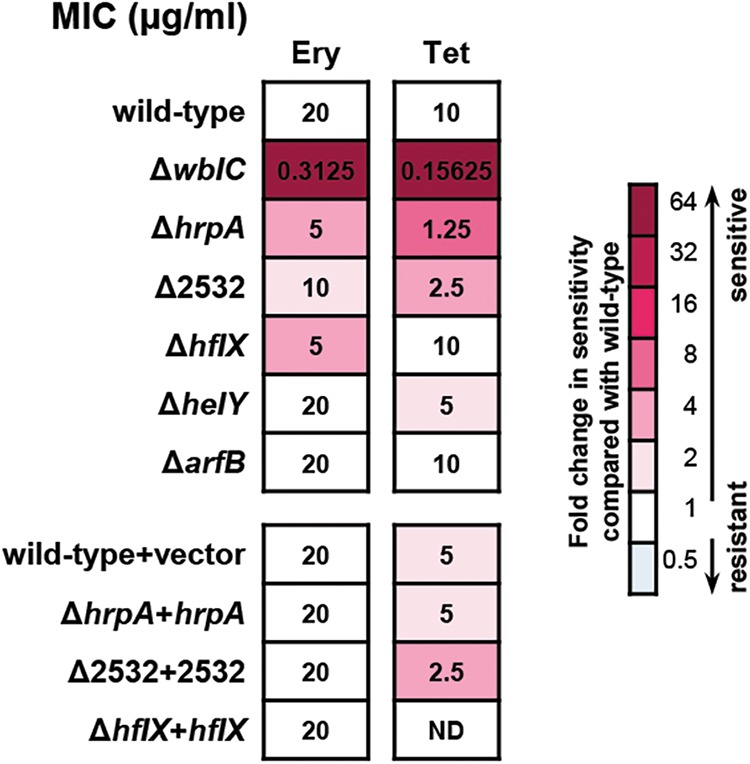
Mutations in WblC-dependent genes encoding ribosome-associated proteins increase sensitivity to translation-targeting antibiotics. MIC assay of wild-type, Δ*wblC*, Δ*hrpA*, Δ*2532*, Δ*hflX*, Δ*arfB*, Δ*helY*, Δ*hrpA*+*hrpA*, Δ*2532 + 2532*, Δ*hflX*+*hflX* strains or wild-type strain with the integrated vector (wild-type+vector) in the presence of translation-targeting antibiotics. MICs were assayed over a range of twofold serial dilutions of erythromycin (Ery) or tetracycline (Tet). The MIC values (in micrograms per milliliter) are indicated in the boxes after 21 h of incubation and represent the median of three independent experiments. The darker background colors indicate that the tested strain is more sensitive to the antibiotic than the wild type. The MICs of the complemented strains were not determined (ND) if there was no difference between median MICs of the wild-type and mutant strains.

Given that several ribosome-associated proteins were enriched during antibiotic stress in a WblC-dependent fashion, we wondered whether newly replaced ribosome-associated proteins have an impact on resistance to translation-targeting antibiotics. To test this, we first compared wild-type and Δ*wblC* strains by determining the MICs of various antibiotics that inhibit different stages of translation (Table S4). We chose erythromycin and tetracycline for further analysis because the Δ*wblC* strain was highly susceptible to those antibiotics (erythromycin [64-fold] and tetracycline [64-fold]) (Fig. 7) compared to other translation-targeting antibiotics (Table S4). Then, we created mutant strains of genes encoding the identified ribosome-associated proteins (*hrpA*, SCO2532, *hflX*, *arfB*, and *helY*) (Table 2) and measured the MICs of two different translation-targeting antibiotics for the wild-type and mutant strains. Among mutant strains lacking ribosome-associated proteins, the strain lacking HrpA (Δ*hrpA*) was fourfold more sensitive to erythromycin and eightfold more sensitive to tetracycline than the wild-type strain was (Fig. 7). Similarly, the strain lacking SCO2532 (Δ*2532*) was twofold more sensitive to erythromycin and fourfold more sensitive to tetracycline than the wild-type strain was (Fig. 7), demonstrating that the ribosome-associated HrpA and SCO2532 proteins indeed contribute to resistance to translation-targeting antibiotics. Interestingly, the strain lacking HflX ribosome-associated GTPase showed a significant effect on sensitivity to erythromycin (fourfold), supporting a previous report that a *hflX* homolog mediates erythromycin resistance (47, 48). Complementation of the *hrpA*, SCO2532, or *hflX* gene restored resistance to erythromycin or tetracycline (except SCO2532 to tetracycline sensitivity), further supporting that these proteins directly mediate antibiotic resistance. Strains lacking HelY or ArfB exhibited a marginal effect (twofold) of sensitivity only to tetracycline (Δ*helY*) or no apparent effect on sensitivity (Δ*arfB*), suggesting that *Streptomyces* might have functionally redundant homologs. In sum, these results demonstrate that ribosome-associated proteins altered by WblC indeed participate in developing resistance to translation-targeting antibiotics.

## DISCUSSION

**FIG 8:**
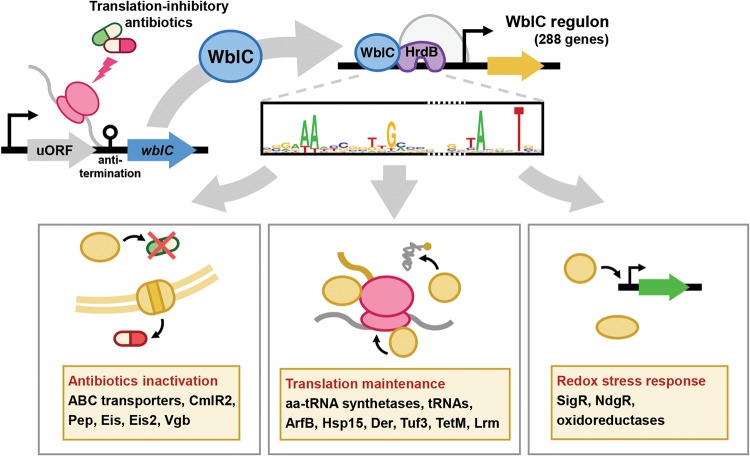
WblC controls genes that are required for counteracting translation-targeting antibiotics. Upon encountering sub-MIC levels of translation-targeting antibiotics, transcription of the *wblC* gene is highly induced by a positive feedback at its autoregulated promoter and upstream ORF (uORF)-mediated transcription attenuation mechanism (16, 17). WblC binds to the conserved sequence (GAAAWY) located 3 nucleotides (nt) upstream of −35 elements in the promoter regions of the WblC-dependent genes and activates transcription of 288 WblC regulon genes by mediating WblC-HrdB cooperative binding to target promoters. The WblC regulon includes genes involved in decreasing antibiotic concentration, inactivating the action of antibiotics, maintaining translation (amino acyl [aa]-tRNA synthetases, tRNAs, alternative elongation factors, and release factors), or resolving redox stress mediated by antibiotics, all of which counteract translation-targeting antibiotics and support growth during antibiotic stress. Degenerate base codes in WblC-activated promoter consensus sequence are as follows: W (A or T), Y (C or T), K (T or G), M (C or A), and N (any nucleotide).

Here, we establish that WblC directly controls 312 genes, comprising 4% of total chromosomal genes in S. coelicolor. Combined analysis of RNA-seq and ChIP-seq data identified such a large number of genes controlled by WblC, illustrating how *Streptomyces* develops antibiotic resistance to translation-targeting antibiotics. These include antibiotic export mediated by CmlR2 and Pep (20, 21), antibiotic inactivation by acetylating drugs (Eis and Eis2) (22, 49) or by linearizing the lactone ring (Vgb) (23), and ribosome protection from antibiotics by dislodging tetracycline from the ribosome (TetM) (25) or methylating rRNA (Lrm) (24) (Fig. 8). Interestingly, the WblC regulon also includes a group of genes that were previously recognized but not documented as antibiotic resistance genes. These are aminoacyl-tRNA synthetases, tRNA processing enzymes, several amino acid biosynthesis enzymes, and tRNAs that are normally required for protein synthesis to support bacterial growth (Table 1). We propose these WblC-activated genes involved in translational maintenance as another type of determinant in intrinsic antibiotic resistance, because WblC activates these genes to allow *Streptomyces* to continue protein synthesis and promote growth at sub-MIC levels of translation-targeting antibiotics (Fig. 5; see also Fig. S4 in the supplemental material), thereby contributing to high levels of intrinsic resistance to translation-targeting antibiotics (Fig. 7). Interestingly, the maintenance of translation speed appears to be mediated by a WblC-induced ribosome remodeling, because WblC indeed alters the composition of 10 ribosome-associated proteins (Fig. 6 and Table 2) that could speed up translation even in the presence of ribosome-targeting antibiotics. Among these WblC-dependent ribosome-associated proteins, ArfB, Hsp15, and HPF were also described as proteins that are required for diverse translational stresses in bacteria (50), which include mRNA truncation-mediated ribosome stalling (ArfB), heat shock-mediated ribosome dissociation (Hsp15), or nutritional down-shift (HPF). The similarity in the protein requirements suggests that stress responses mediated by sub-MIC levels of antibiotics are physiologically similar to those mediated by other translational stress responses affecting protein synthesis and bacterial growth rate. Finally, the WblC-mediated ribosomal alteration indeed contributes to resistance to translation-targeting antibiotics, because strains lacking the ribosome-associated proteins (HrpA, SCO2532, and HflX) that were enriched in a *wblC*-dependent and tetracycline-dependent manner are more sensitive to several translation-targeting antibiotics than the wild-type strain (Fig. 7).

The genus *Streptomyces* often contains a large number of paralogous genes, whose biological functions are not well understood. Transcription profiles of paralogous genes involved in translation suggest that the requirements of those genes may differ during antibiotic stress. For instance, *alaS2* (SCO7600) and *alaS* (SCO1501) are paralogous genes encoding Ala-tRNA synthetases, and expression of the former is dependent on WblC and tetracycline but expression of the latter is not (Fig. S5 and Table S2). Similar patterns of transcription profiles among the WblC-activated genes and their paralogs were observed, including paralogs of Trp-tRNA synthetase (*trpS* [SCO3334] and *trpS2* [SCO4839]), rRNA methyltransferase (*lrm* [SCO6089] and *ksgA* [SCO3149]), release factor ArfB/RF-2 (*arfB* [SCO4278] and SCO2972), ribosome biogenesis GTPase RsgA/EngC (SCO6149 and SCO5211), EF-Tu (*tuf3* [SCO1321] and *tuf1* [SCO4662]), and EF-G (*fusB* [SCO6589], *tetM* [SCO0783], *fusA* [SCO4661], and SCO1528) (Fig. S5 and Table S2). Also, elevated mRNA levels of the WblC-activated paralogs upon tetracycline treatment seem to correlate with their proposed function that maintains translation at sub-MIC levels of antibiotics, because one of the WblC-activated paralogs, ArfB (SCO4278), was indeed enriched in the 70S ribosomal fractions during antibiotic stress (Fig. 6 and Table 2).

10.1128/mBio.00625-20.6 FIG S5 mRNA levels of translation-related paralogs are altered upon tetracycline treatment. Normalized read counts from wild-type cells untreated (-) or treated (+) with 2 μg/ml tetracycline were plotted for each gene. Paralogous genes with a similar predicted function are grouped and indicated below. WblC-dependent genes among paralogs are plotted in magenta or blue and indicated in bold text. Locus tags of each gene are shown in parentheses as follows: *alaS2* (SCO7600), *alaS* (SCO1501), *trpS* (SCO3334), *trpS2* (SCO4839), *thrS2* (SCO3778), *thrS* (SCO1531), *lrm* (SCO6089), *ksgA* (SCO3149), *arfB* (SCO4278), *prfB* (SCO2972), *tuf3* (SCO1321), *tuf1* (SCO4662), *fusB* (SCO6589), *tetM* (SCO0783), and *fusA* (SCO4661). Download FIG S5, PDF file, 0.3 MB. Copyright © 2020 Lee et al. 2020 Lee et al. This content is distributed under the terms of the Creative Commons Attribution 4.0 International license.

Previous studies reported that large numbers of genes were induced or repressed by subinhibitory concentrations of antibiotics in other bacteria (51, 52). Such a large alteration in RNA profiles affects various aspects of bacterial physiology that eventually lead to cellular adaptation or resistance to antibiotics. However, it has not clearly identified a specific set of genes or regulators responsive to each antibiotic mostly because a large number of genes were overlapped independent of antibiotics and appeared to involve multiple signaling pathways (52). WblC is a unique example in a sense that, even though it is a single transcription regulator, it is activated by sub-MIC levels of antibiotics and directly controls more than 300 genes, thereby mediating intrinsic resistance to such a wide range of antibiotics (9). Interestingly, WblC represses mRNA levels of 24 genes, suggesting that WblC also functions as a transcriptional repressor. Similar examples could be found in other WhiB-like (Wbl) family proteins. In *Streptomyces*, WhiB and WhiA corepress transcription of *filP*, which is required for the cessation of aerial hyphae at the developmental stage (53). Likewise, Mycobacterium tuberculosis WhiB1 represses its own transcription and transcription of *groES2* encoding an essential chaperone by interacting with SigA sigma factor (54–56), suggesting that this could be one of the common features of WhiB-like (Wbl) family proteins. However, the functional role or physiological relevance of WblC-repressed genes is presently unclear because they are not grouped as specific functional categories.

Overall, our findings that WblC controls a large number of genes in response to antibiotics, including the newly discovered translation maintenance genes, illuminate how bacteria develop intrinsic antibiotic resistance by altering their physiology in response to antibiotics. Further research is needed to understand the underlying mechanism of intrinsic resistance, which may lead to an alternative way to treat antibiotic-resistant bacteria such as M. tuberculosis.

## MATERIALS AND METHODS

### Strains, growth conditions, and reagents.

Streptomyces coelicolor strains and DNA primers used in this study are listed in Table S1 in the supplemental material. Spores of each strain, prepared according to standard procedures (4), were inoculated in YEME liquid medium containing 5 mM MgCl_2_ and 10% sucrose and grown at 30°C with shaking at 180 rpm. For antibiotic stress conditions, a freshly made solution of tetracycline hydrochloride (Sigma) or stock solutions of chloramphenicol (Sigma) or erythromycin A dihydrate (Sigma) at the indicated concentrations were treated to early exponential cells (optical density at 600 nm [OD_600_] of 0.1 to 0.5). For determining MIC, erythromycin (Sigma), tetracycline hydrochloride (Sigma), lincomycin hydrochloride (Fluka), chloramphenicol (Sigma), fusidic acid sodium salt (Sigma), hygromycin B concentrated solution (Duchefa), linezolid (Sigma), streptomycin sulfate salt (Sigma), thiostrepton from Streptomyces azureus (Sigma), puromycin dihydrochloride from Streptomyces alboniger (Sigma), and spectinomycin dihydrochloride pentahydrate (Fluka) were used. E. coli strains BW25113/pIJ790 and ET12567/pUZ8002 were grown and used as previously described (57), and E. coli DH5α was grown in LB medium for standard plasmid manipulations.

10.1128/mBio.00625-20.7 TABLE S1 List of S. coelicolor strains and primers used in this study. Download Table S1, PDF file, 0.3 MB. Copyright © 2020 Lee et al. 2020 Lee et al. This content is distributed under the terms of the Creative Commons Attribution 4.0 International license.

10.1128/mBio.00625-20.8 TABLE S2 WblC regulon genes. Download Table S2, PDF file, 0.3 MB. Copyright © 2020 Lee et al. 2020 Lee et al. This content is distributed under the terms of the Creative Commons Attribution 4.0 International license.

10.1128/mBio.00625-20.9 TABLE S3 Intergenic small RNA and antisense RNA targets of WblC. Download Table S3, PDF file, 0.2 MB. Copyright © 2020 Lee et al. 2020 Lee et al. This content is distributed under the terms of the Creative Commons Attribution 4.0 International license.

10.1128/mBio.00625-20.10 TABLE S4 Minimum inhibitory concentrations (MICs) of various antibiotics. Download Table S4, PDF file, 0.3 MB. Copyright © 2020 Lee et al. 2020 Lee et al. This content is distributed under the terms of the Creative Commons Attribution 4.0 International license.

### Translation rate measurement by *in vivo* [^35^S]Met/Cys pulse-chase incorporation.

Cells were grown to an OD_600_ of approximately 0.4 and treated with tetracycline or chloramphenicol at the indicated concentrations for 1 h. Then, cells were normalized to an OD_600_ of 0.2 by adding liquid medium. Aliquots (500 μl) from the normalized cultures were used for protein labeling. One microcurie of EasyTag Express ^35^S protein labeling mix (PerkinElmer) was added to the aliquots and incubated at 30°C for 10 min for pulse-labeling; 0.5 mg of cold methionine was added to chase, and the sample was incubated for an additional 5 min. Cells were washed twice with phosphate-buffered saline (PBS), resuspended in 5 μl PBS, and dot blotted onto Whatman filter paper. An autoradiograph was taken after overnight exposure and quantified with Multi Gauge v3.0 (Fujifilm).

### Determining ribosome composition by liquid chromatography-tandem mass spectrometry.

Wild-type and Δ*wblC* cells were grown to an OD_600_ of approximately 0.4 and treated with 0.25 μg/ml tetracycline for 2 h. Control cells were grown in parallel without tetracycline (final OD between 0.7 and 1.0). Then, the cells were harvested and washed with wash buffer (20 mM Tris-Cl [pH 7.4], 100 mM NaCl, 10 mM MgCl_2_) and were lysed by sonication in lysis buffer (20 mM Tris-Cl [pH 7.4], 200 mM NH_4_Cl, 10 mM MgCl_2_, 0.5 mM EDTA, 6 mM β-mercaptoethanol) with 5 U/ml RNase inhibitor (Applied Biosystems) and 1 mM phenylmethylsulfonyl fluoride (PMSF). After 5 U/ml Turbo DNase (Ambion) treatment at 4°C for 15 min, lysates were clarified twice by centrifugation (22,000 × g, 10 min, 4°C). Crude ribosomes were pelleted by ultracentrifugation (100,000 × g, 1 h, 4°C), rinsed with lysis buffer by gentle swirling, and resuspended in lysis buffer with 10 U/ml RNase inhibitor on ice. Crude ribosomes were loaded onto a 5 to 30% sucrose gradient prepared with lysis buffer and spun at 150,000 × g for 2.5 h. The 70S fractions were identified by measuring absorbance at 254 nm and pooled. Integrity of rRNA was checked using standard formaldehyde agarose gel electrophoresis for all biologically independent replicates. The purified ribosome samples were denatured with 8 M urea and reduced with 10 mM dithiothreitol (DTT). The cysteine thiol groups were alkylated with 40 mM iodoacetamide in the dark. The samples were diluted to 1 M urea and digested with trypsin (1:50 ratio [wt/wt]) overnight at 37°C. The tryptic digests were cleaned up using a C_18_-SPE column (Supelco). The samples were evaporated using a speed-vac and resuspended in 25 mM ammonium bicarbonate buffer. The final peptides were quantified using the bicinchoninic acid (BCA) assay, and then 2 μg of each sample was subject to liquid chromatography-tandem mass spectrometry (LC-MS/MS) analysis. LC-MS/MS experiments with higher-energy collisional dissociation (HCD) fragmentation mode were performed using Q-Exactive Orbitrap mass spectrometry (Thermo Fisher Scientific) coupled with nanoACQUITY UPLC (Waters), equipped with an in-house-packed capillary trap column (150-μm inner diameter, 3 cm long) and analytical column (75-μm inner diameter, 100 cm long) with 3-μm Jupiter C_18_ particles (Phenomenex) at a flow rate of 300 nl/min. A linear gradient (100 min) was applied for each biological replicate. Acquired data sets were analyzed by MaxQuant (v1.5.3.30) (58) with the Andromeda search engine at 10 ppm precursor ion mass tolerance against Swiss-Prot S. coelicolor database at <1% of protein false-discovery rate (FDR). The label-free quantification mode was applied to the quantitative analysis of ribosome-associated proteins. Statistical analysis of experiments was performed using Perseus (59), which accompanies MaxQuant. Proteins were considered significantly different when FDR-adjusted *P* values were <0.05 by the two-tailed Student’s *t* test.

### Determining MIC.

MICs were determined using the resazurin assay (8) with the following modifications. A total of 10^6^ spores/ml were inoculated in YEME medium, and a 150-μl aliquot of the inoculum was transferred to each well in 96-well plates. Then, the inoculum was treated with twofold serial dilutions of erythromycin, tetracycline, lincomycin, chloramphenicol, fusidic acid, hygromycin B, linezolid, streptomycin, thiostrepton, puromycin, and spectinomycin and grown for 21 h at 30°C with shaking at 180 rpm. Fifteen microliters of 0.03% resazurin (Fluka) was added to each well, and cells were grown for another 1 h. MICs were determined by measuring the absorbance at 570 nm and 600 nm and then calculating the *A*_570_/*A*_600_ ratio. If the *A*_570_/*A*_600_ ratio was <1.2 (the value of no-growth control), we considered that growth was significantly inhibited, and the MIC was determined as the lowest concentration that exhibits an *A*_570_/*A*_600_ ratio of <1.2.

### Data availability.

RNA-seq and ChIP-seq data have been deposited in the Gene Expression Omnibus (GEO) with the accession number GSE136168. LC-MS/MS data have been deposited in the Proteomics Identifications (PRIDE) archive with the accession code PXD015538. Genome sequences and annotations for S. coelicolor A3(2) reference genome (accession code NC_003888.3) are available from the National Center for Biotechnology Information (NCBI).

10.1128/mBio.00625-20.1 TEXT S1 Supplemental methods. Download Text S1, PDF file, 0.4 MB. Copyright © 2020 Lee et al. 2020 Lee et al. This content is distributed under the terms of the Creative Commons Attribution 4.0 International license.
