# Supplementary material for: The WblC/WhiB7 Transcription Factor Controls Intrinsic Resistance to Translation-Targeting Antibiotics by Altering Ribosome Composition
Source: mBio. 2020 Apr 14;11(2):e00625-20. doi: 10.1128/mBio.00625-20 (PMC7157823; doi:10.1128/mBio.00625-20)
Supplement: FIG S1 [file mBio.00625-20-sf001.pdf]

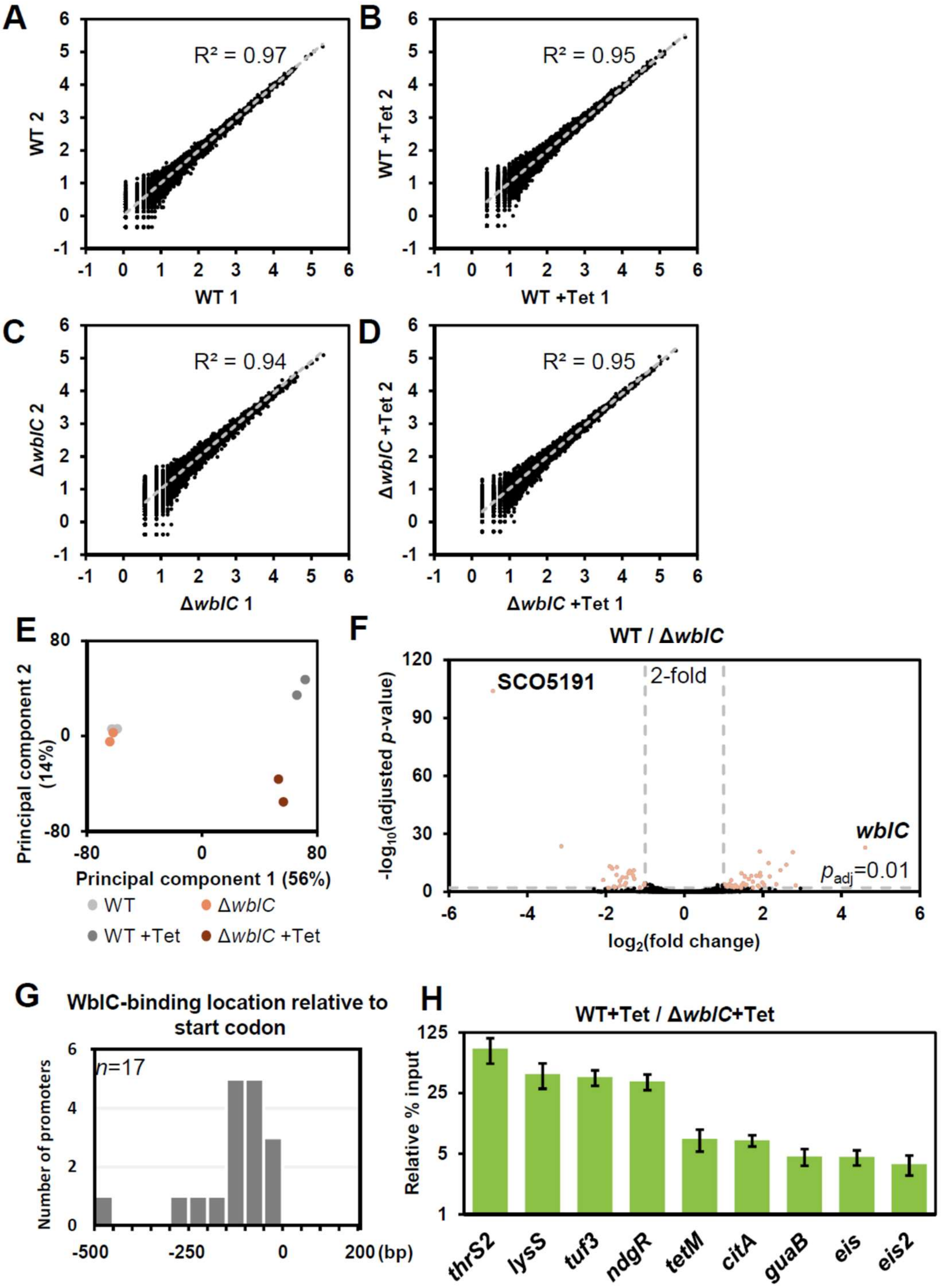

Figure S1. RNA-seq sample statistics. (A-D) Scatter plots of [$\log_{10}$(normalized count)] between biological duplicates of untreated wild-type (A), tetracycline-treated wild-type (B), untreated Δ*wblC* (C), and tetracycline-treated Δ*wblC* (D) strains. Genes excluding pseudogenes and rRNA genes were plotted. Linear regression analysis between biological duplicates showed the coefficient of determination (R^2) values of 0.97 (A), 0.95 (B), 0.94 (C), and 0.95 (D), suggesting that RNA-seq results are highly reproducible. (E) Principal component analysis of the 8 RNA-seq samples. The fractions of variance explained by each component are denoted on each axis. It resulted in three distinct clusters among untreated strains, tetracycline-treated wild-type, and tetracycline-treated

Δ*wblC* strains. (F) Fewer genes are affected by *wblC* deletion in the absence of tetracycline. Volcano plot comparing RNA-seq profiles of untreated wild type and Δ*wblC* strains. Volcano plot comparing RNA-seq profiles of wild type and ΔwblC in the absence of tetracycline. DEGs (pink) are defined by the adjusted *p*-value (p_{adj}<0.01, horizontal grey dashed line) and fold-change cut-off (|$\log_2$[fold change]|>1, vertical grey dashed lines). The *wblC* gene exhibiting decreased expression in the Δ*wblC* strain and the SCO5191 gene exhibiting increased expression in the Δ*wblC* strain are indicated. (G) Distribution of WblC-binding summits among the WblC-repressed genes. WblC binding summits are indicated as the location relative to the start codon of the first gene in each operon. The total number of promoters (n) is indicated. (H) WblC's binding to selected promoters is confirmed by ChIP-qPCR. The ChIP-qPCR assay was performed in wild-type and Δ*wblC* strains treated with 2 μg/ml tetracycline (Tet) for 1 h. Relative % input represents [% input in wild-type/% input in Δ*wblC*] in the presence of tetracycline. Shown are means ±SE from three independent experiments ($p<0.05$).
