# Supplementary material for: The WblC/WhiB7 Transcription Factor Controls Intrinsic Resistance to Translation-Targeting Antibiotics by Altering Ribosome Composition
Source: mBio. 2020 Apr 14;11(2):e00625-20. doi: 10.1128/mBio.00625-20 (PMC7157823; doi:10.1128/mBio.00625-20)
Supplement: FIG S2 [file mBio.00625-20-sf002.pdf]

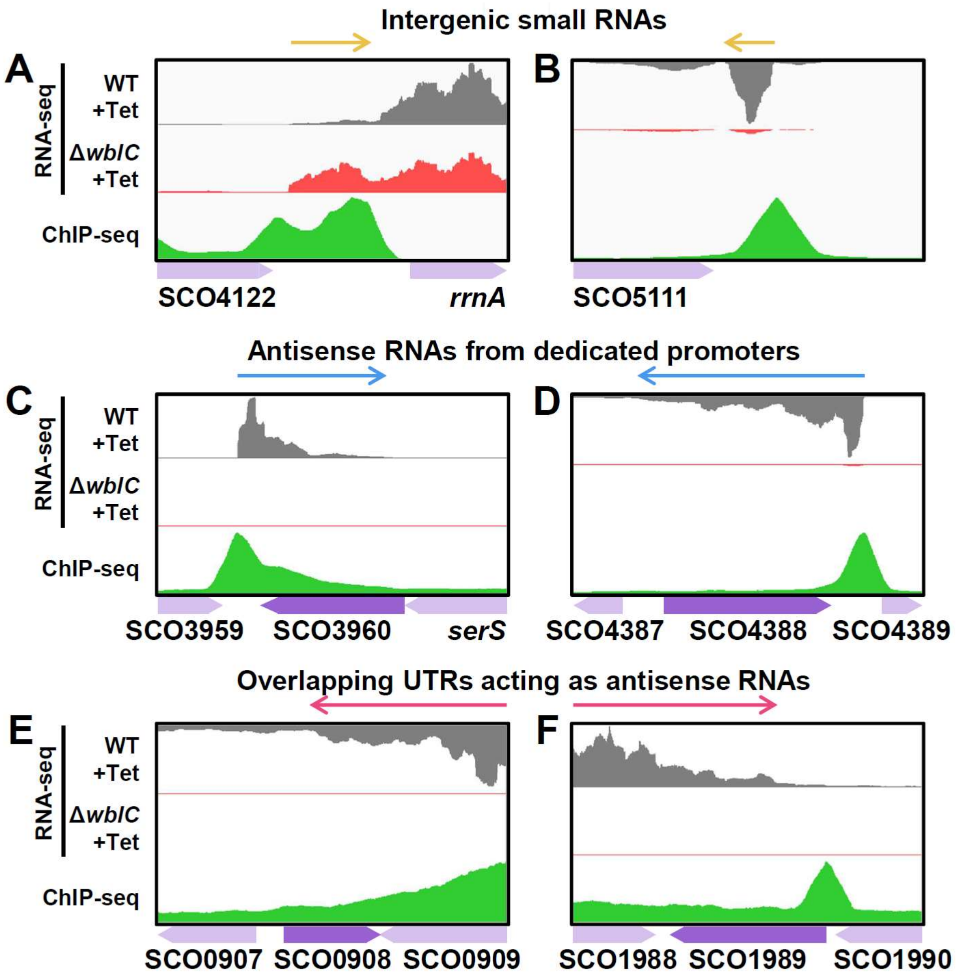

Figure S2. WblC controls noncoding RNAs and antisense RNAs. (A and B) Examples of noncoding RNAs located in the intergenic region between SCO4122 and *rrnA* (A) or downstream of SCO5111 (B). (C and D) Examples of antisense RNAs with independent promoters overlapping with SCO3960 (C) or SCO4388 (D). (E and F) Examples of antisense RNAs originated from UTRs of the neighboring SCO0909 (E) and SCO1988 (F) genes.
