# Supplementary material for: The WblC/WhiB7 Transcription Factor Controls Intrinsic Resistance to Translation-Targeting Antibiotics by Altering Ribosome Composition
Source: mBio. 2020 Apr 14;11(2):e00625-20. doi: 10.1128/mBio.00625-20 (PMC7157823; doi:10.1128/mBio.00625-20)
Supplement: FIG S3 [file mBio.00625-20-sf003.pdf]

Position of the last T nucleotide in the -10 motifs relative to TSS

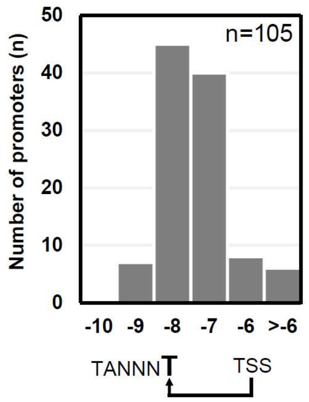

Figure S3. The position of -10 motifs relative to TSS. The promoters (n=105) with identified –10 motifs were grouped according to the position relative to TSS. When -10 motif and reported TSS are >50 bp apart, those –10 motifs were excluded from the analysis.
