# Supplementary material for: The WblC/WhiB7 Transcription Factor Controls Intrinsic Resistance to Translation-Targeting Antibiotics by Altering Ribosome Composition
Source: mBio. 2020 Apr 14;11(2):e00625-20. doi: 10.1128/mBio.00625-20 (PMC7157823; doi:10.1128/mBio.00625-20)
Supplement: FIG S4 [file mBio.00625-20-sf004.pdf]

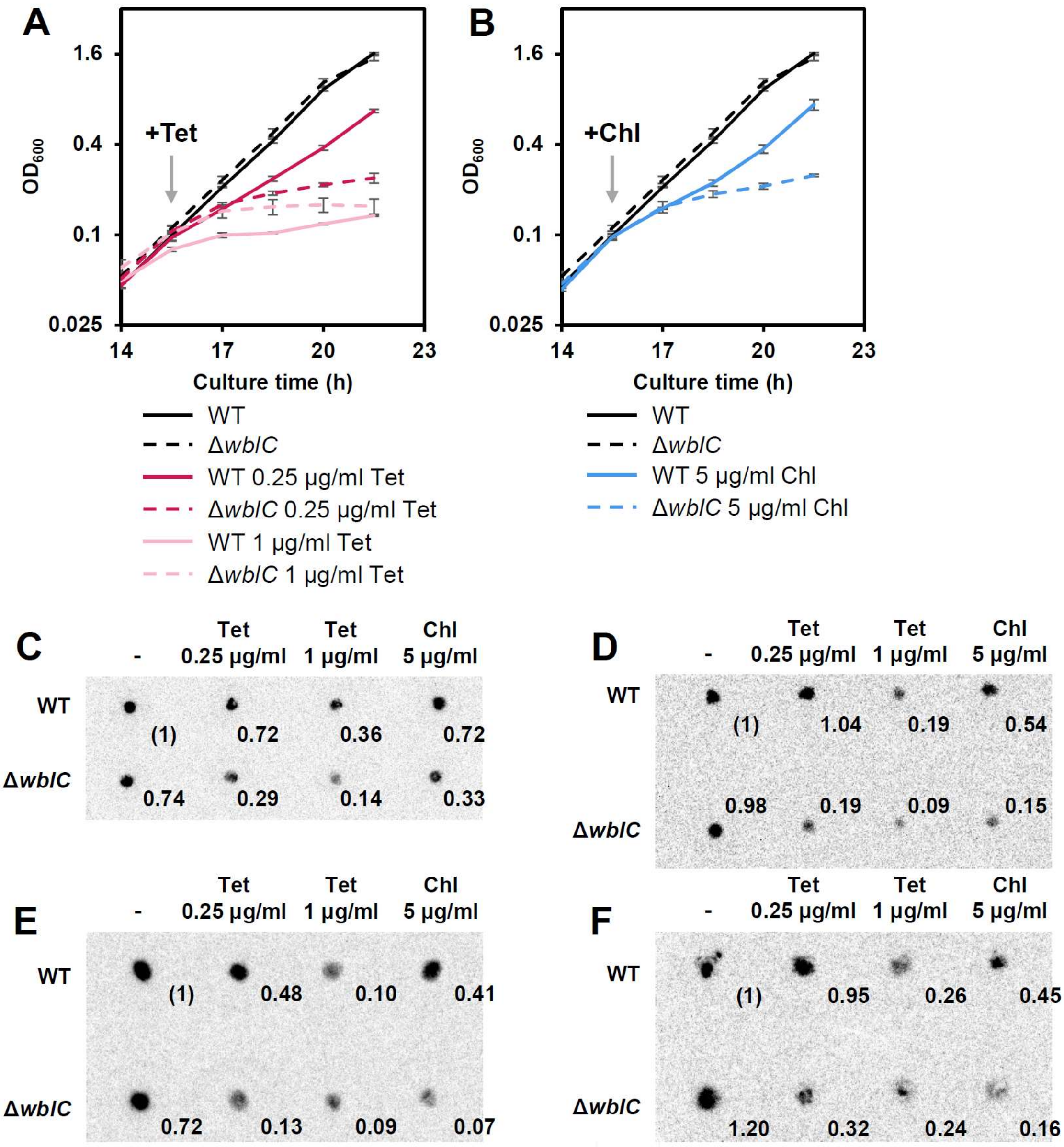

Figure S4. WblC affects *Streptomyces* growth and translation rate at sub-MIC levels of antibiotics. (A-B) Growth curves of wild-type and Δ*wblC* strains in YEME liquid medium untreated or treated with tetracycline (A) or chloramphenicol (B) at indicated concentrations. Bacteria were grown at 37 °C for 15.5 h with shaking after spore inoculation. After antibiotics were treated at 15.5 h (indicated by arrows), growth was monitored for additional 6 h. Shown are mean ±SE from three independent experiments. (C-F) Autoradiographs of four independent experiments of *in vivo* ^{35}S-Met/Cys pulse-chase labeling, related to Fig. 5. Signal intensities of untreated wild-type were set to 1 and compared to others as relative ratios.
