# Supplementary material for: The WblC/WhiB7 Transcription Factor Controls Intrinsic Resistance to Translation-Targeting Antibiotics by Altering Ribosome Composition
Source: mBio. 2020 Apr 14;11(2):e00625-20. doi: 10.1128/mBio.00625-20 (PMC7157823; doi:10.1128/mBio.00625-20)
Supplement: FIG S5 [file mBio.00625-20-sf005.pdf]

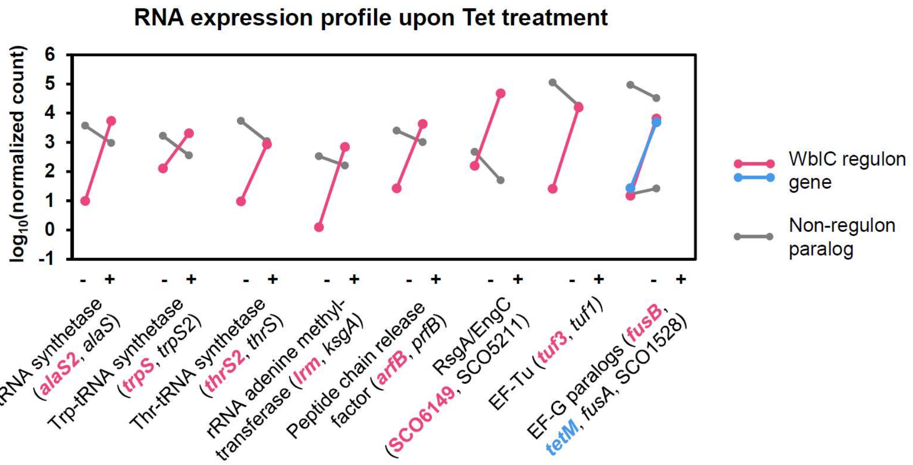

Figure S5. mRNA levels of translation-related paralogs are altered upon tetracycline treatment. Normalized read counts from wild-type cells untreated (-) or treated (+) with 2 μg/ml tetracycline were plotted for each gene. Paralogous genes with a similar predicted function are grouped and indicated below. WblC-dependent genes among paralogs are plotted in magenta or blue and indicated in bold text. Locus tags of each gene are as follows: *alaS2* (SCO7600), *alaS* (SCO1501), *trpS* (SCO3334), *trpS2* (SCO4839), *thrS2* (SCO3778), *thrS* (SCO1531), *lrm* (SCO6089), *ksgA* (SCO3149), *arfB* (SCO4278), *prfB* (SCO2972), *tuf3* (SCO1321), *tuf1* (SCO4662), *fusB* (SCO6589), *tetM* (SCO0783), and *fusA* (SCO4661).
