# Supplementary material for: The WblC/WhiB7 Transcription Factor Controls Intrinsic Resistance to Translation-Targeting Antibiotics by Altering Ribosome Composition
Source: mBio. 2020 Apr 14;11(2):e00625-20. doi: 10.1128/mBio.00625-20 (PMC7157823; doi:10.1128/mBio.00625-20)
Supplement: TABLE S1 [file mBio.00625-20-st001.pdf]

Table S1. List of *S. coelicolor* strains and primers used in this study.

A. Strains

Name	Genotype	Reference
M145 (wild-type)	SCP1⁻ SCP2⁻	(1)
M145+pSET162	M145::pSET162	(2)
Δ*wblC*	M145 *wblC*::*aac(3)IV*	(3)
Δ2532	M145 ΔSCO2532	This paper
Δ*hrpA*	M145 *hrpA*::*aac(3)IV*	This paper
Δ*hflX*	M145 *hflX*::*aac(3)IV*	This paper
Δ*arfB*	M145 *arfB*::*aac(3)IV*	This paper
Δ*helY*	M145 *helY*::*aac(3)IV*	This paper
Δ2532+2532	M145::pSET162::SCO2532 ΔSCO2532	This paper
Δ*hrpA*+*hrpA*	M145::pSET162::*hrpA* *hrpA*::*aac(3)IV*	This paper
Δ*hflX*+*hflX*	M145::pSET162::*hflX* *hflX*::*aac(3)IV*	This paper

B. Primers

Name	Sequence (5' to 3')
**Primers used for ChIP-qPCR**	
tuf3 ChIP F	cattcgacgtgcgacgaagcg
tuf3 ChIP R	ggtgaagtgggcgacggca
tetM ChIP F	catgatggcgccgtccgaac
tetM ChIP R	ggaaggggcgctgggggaa
cvnA1 ChIP F	cttctacgtccggtgatccg
cvnA1 ChIP R	gcacagtgcaggatctccaac
SCO4914 ChIP F	ggtggtcatgctgccgattg
SCO4914 ChIP R	gggcatggtcaccagacgag
guaB2 ChIP F	gggattatcggccacccgtcat
guaB2 ChIP R	gctgcacatcccagtcgatcag
SCO3064 ChIP F	tccgcgtctcaagtccttcc
SCO3064 ChIP R	cgtcgaactgtcgccaaagg
wblE ChIP F	gagtctcttcttggcgatcggg
wblE ChIP R	gcgtggtcctgtggtttgaaga
citA ChIP F	caaacgagtcggaaaggtcacacag
citA ChIP R	ctcggggtcgaccatcggg
thrS2 ChIP F	gcagtaccgtcgtccccatg
thrS2 ChIP R	gtcagcagtcagcgcgcc
lysS ChIP F	gcggttttcgcggatgcgg
lysS ChIP R	acttccccggaaccgctcc
ndgR ChIP F	gagacgggagtatcgtgtcatgg
ndgR ChIP R	ccgggccggactccagag
guaB ChIP F	ccgttagcatggacatccgcac
guaB ChIP R	gtcgtaggtcagcccgagtgtc
eis ChIP F	ggagccgggcaggcgatc
eis ChIP R	gttccagtcggggaactcggc
eis2 ChIP F	gtgccgccctggtacgcat
eis2 ChIP R	acccgcggctccatcgtcat
**Primers used for mutant strain construction**	

SCO2532 up HindIII F	gcacAAgcTttgacttcgctcacc
SCO2532 up BamHI R	tcatggATcCgcgctctaggcctgc
SCO2532 down BamHI F	cagcGGATccatgtcgatcgacgtc
SCO2532 down EcoRI R	cgacgaaTTcccggcgatcggga
hrpA disrupt F	ccaggtttttcccgaggatgagatcctggaacccgtatgATTCCGGGGATCCGTCGACC
hrpA disrupt R	ggccgccggtcgaactcaccctccgtacgggcctcgtcaTGTAGGCTGGAGCTGCTTC
hflX disrupt F	gccgcgccgacccсttcccagctacgtaaggatccaatgATTCCGGGGATCCGTCGACC
hflX disrupt R	ctcggtcggtcggcctgctgttcggccgcgcgggtctcaTGTAGGCTGGAGCTGCTTC
arfB disrupt F	atattcgagtgacgcgggccgcccgcggacgggaacatgATTCCGGGGATCCGTCGACC
arfB disrupt R	cgtactggagctggactgagacgcgggtccttccggtcaTGTAGGCTGGAGCTGCTTC
helY disrupt F	cgcccccacggaacccccacacggccgatcccgataatgATTCCGGGGATCCGTCGACC
helY disrupt R	actcccgccgggccgggtgaggtgtcggggccggggtcaTGTAGGCTGGAGCTGCTTC
**Primers used for gene complementation**	
SCO2532 AseI F	cgaccatTaATgcatcgcctacc
SCO2532 XbaI R	tcgtTctAgActacttccccttgg
hrpA NotI F	tggagCgGCcgccaccctgtcaag
hrpA XbaI R	accctcTAGacgggcctcgtcacg
hflX NotI F	tgggGcgGccgcaaccaggacgg
hflX XbaI R	gggcTctAgAtcggtcggcctgctg
**Primers used for genotype confirmation**	
SCO2532 seq F	accggtcggataaacccctcg
SCO2532 seq R	tggatgtgcagttgctccatgg
hrpA seq F	ctgaaaagcgataggtcgccgg
hrpA seq R	gctttttctgcgttgcgctgc
hflX seq F	gagaaggccgcggggaagcag
hflX seq R	ggtcagtgacccgtacaggc
arfB seq F	ctaccaccatgaacgtcgagag
arfB seq R	ggacccgagaacttctacctgg
helY seq F	acgacgtgacctgaggaccg
helY seq R	tgcgcacagagcgcggaagg
attB seq F	atgcccgccgtgaccgtcgagaacccgctg
attB seq R	gttggtgatggtgccgccgccaccgttgga
attP seq R	cccagggcgagcaattccgagaca

References

1. Kieser T, Bibb MJ, Buttner MJ, Chater KF, Hopwood DA. 2000. Practical *Streptomyces* Genetics. John Innes Foundation, Norwich.
2. Shin JH, Singh AK, Cheon DJ, Roe JH. 2011. Activation of the SoxR regulon in *Streptomyces coelicolor* by the extracellular form of the pigmented antibiotic actinorhodin. J Bacteriol 193:75-81.
3. Fowler-Goldsworthy K, Gust B, Mouz S, Chandra G, Findlay KC, Chater KF. 2011. The actinobacteria-specific gene *wblA* controls major developmental transitions in *Streptomyces coelicolor* A3(2). Microbiology 157:1312-28.
