# Supplementary material for: The WblC/WhiB7 Transcription Factor Controls Intrinsic Resistance to Translation-Targeting Antibiotics by Altering Ribosome Composition
Source: mBio. 2020 Apr 14;11(2):e00625-20. doi: 10.1128/mBio.00625-20 (PMC7157823; doi:10.1128/mBio.00625-20)
Supplement: TABLE S2 [file mBio.00625-20-st002.pdf]

Table S2. WblC regulon genes.

Locus tag	RNA-seq (WT+Tet/Δ*wblC*+Tet)		First gene of operon	ChIP-seq peak		Gene name	Product	Category (WblC-activated gene only)	Sub-category (WblC-activated gene only)
	Fold change	-log10 (adjusted *p*-value)		Summit location	Fold enrichment				
SCO0105	10.4	16.0	SCO0107	-	-	xlnC	endo-1%2C4-beta-xylanase	6. Hydrolases	-
SCO0107	56.2	65.9	SCO0107	89991	11.7	-	aminoglycoside nucleotidyltransferase	3. Transferases	-
SCO0165	11.4	23.2	SCO0165	156074	3.8	-	hypothetical protein	-	-
SCO0408	218.4	94.3	SCO0408	428678	14.1	-	methyltransferase	3. Transferases	3B. Methyltransferases
SCO0500	9.4	67.1	SCO0500	533777	8.1	-	hypothetical protein	-	-
SCO0527	5.1	25.5	SCO0527	559123	21.3	scoF	cold shock protein	-	-
SCO0783	179.5	151.4	SCO0783	829817	4.3	tetM	tetracycline resistance protein	2. P-loop NTPases	2D. GTPases
SCO0784	209.2	35.0	SCO0783	-	-	-	hypothetical protein	-	-
SCO0786	9.9	3.6	SCO0783	-	-	-	hypothetical protein	4. Integral membrane proteins	-
SCO0795	78.8	141.1	SCO0795	841851	9.7	-	hypothetical protein	-	-
SCO0796	59.1	119.5	SCO0795	-	-	-	hypothetical protein	4. Integral membrane proteins	-
SCO0797	5.2	18.5	SCO0795	-	-	-	hypothetical protein	-	-
SCO0798	4.7	7.0	SCO0795	-	-	-	hypothetical protein	-	-
SCO0854	129.9	25.2	SCO0854	899438	20.8	-	hypothetical protein	-	-
SCO0906	6.6	11.8	SCO0909	-	-	-	hypothetical protein	5. Oxidoreductases	-
SCO0907	11.0	27.4	SCO0909	-	-	-	dehydrogenase	5. Oxidoreductases	-
SCO0909	460.4	125.9	SCO0909	954312	8.9	-	hypothetical protein	-	-
SCO1008	2.1	4.5	SCO1008	1063183	4.1	-	hypothetical protein	7. Transcription regulators	-
SCO1028	8.2	42.5	SCO1028	1087009	5.3	-	lyase	-	-
SCO1143	9.7	19.3	SCO1144	-	-	mltG	hypothetical protein	4. Integral membrane proteins	-
SCO1144	12.7	55.8	SCO1144	1204362	16.0	-	ABC transporter ATP-binding protein	2. P-loop NTPases	2A. ABC transporters with transmembrane domains
SCO1147	150.8	140.7	SCO1147	1205844	19.5	-	ABC transporter transmembrane subunit	2. P-loop NTPases	2A. ABC transporters with transmembrane domains
SCO1148	139.5	71.6	SCO1147	-	-	-	ABC transporter	2. P-loop NTPases	2A. ABC transporters with transmembrane domains
SCO1149	3.1	6.7	SCO1147	-	-	-	hypothetical protein	-	-
SCO1151	2.7	11.5	SCO1151	1211461	2.7	-	hypothetical protein	4. Integral membrane proteins	-
SCO1305	30.4	22.2	SCO1307	-	-	-	hypothetical protein	-	-
SCO1306	35.3	46.6	SCO1307	-	-	-	hypothetical protein	2. P-loop NTPases	2C. Other ATPases
SCO1307	38.7	98.0	SCO1307	1385325	23.5	-	hypothetical protein	-	-
SCO1320	32.9	95.5	SCO1321	-	-	-	hypothetical protein	3. Transferases	3B. Methyltransferases
SCO1321	99.6	241.6	SCO1321	1397589	23.1	tuf3	elongation factor Tu	2. P-loop NTPases	2D. GTPases
SCO1340	56.2	124.7	SCO1340	1417164	3.2	-	hypothetical protein	-	-
SCO1341	35.8	13.8	SCO1340	-	-	-	lipoprotein	-	-
SCO1360	5.0	10.7	SCO1362	-	-	-	GntR family transcriptional regulator	7. Transcription regulators	-
SCO1361	5.3	13.8	SCO1361	1439065	2.8	-	hypothetical protein	-	-
SCO1362	27.1	67.5	SCO1362	1440995	12.1	-	hypothetical protein	4. Integral membrane proteins	-
SCO1404	2.5	2.4	SCO1406	-	-	-	hypothetical protein	-	-
SCO1405	4.4	9.4	SCO1406	-	-	-	HSP90 family protein	-	-
SCO1406	4.6	30.0	SCO1406	1496959	4.2	-	hypothetical protein	-	-
SCO1413	26.4	103.7	SCO1413	1509449	7.5	-	hypothetical protein	-	-
SCO1448	11.6	60.0	SCO1448	1544738	2.2	-	transporter	4. Integral membrane proteins	4A. Major facilitator superfamily
SCO1450	2.5	9.7	SCO1448	-	-	-	uracyl permease	4. Integral membrane proteins	-
SCO1456	22.2	18.6	SCO1457	-	-	-	hypothetical protein	-	-
SCO1457	18.7	88.2	SCO1457	1555587	3.5	-	transporter	4. Integral membrane proteins	4A. Major facilitator superfamily
SCO1502	2.1	4.3	SCO1503	-	-	-	hypothetical protein	-	-
SCO1503	2.8	12.8	SCO1503	1606615	8.6	-	hypothetical protein	4. Integral membrane proteins	-
SCO1508	3.5	16.5	SCO1509	-	-	hisS	histidyl-tRNA synthetase	1. aminoacyl-tRNA synthesis & editing enzymes	-
SCO1509	5.2	32.0	SCO1509	1614186	14.1	-	hydrolase	6. Hydrolases	-
SCO1545	26.9	71.5	SCO1545	1654146	2.9	-	acetyltransferase	3. Transferases	3A. GNATs
SCO1594	2.6	11.2	SCO1595	-	-	pheT	phenylalanyl-tRNA synthetase subunit beta	1. aminoacyl-tRNA synthesis & editing enzymes	-
SCO1595	4.6	39.0	SCO1595	1708351	15.3	pheS	phenylalanyl-tRNA synthetase subunit alpha	1. aminoacyl-tRNA synthesis & editing enzymes	-
SCO1624	114.2	69.4	SCO1624	1738983	3.2	-	acetyltransferase	3. Transferases	3A. GNATs
SCO1631	5.1	33.5	SCO1631	1747869	4.3	helY	helicase	2. P-loop NTPases	2C. Other ATPases
SCO1672	8.4	42.5	SCO1672	1794029	8.6	-	hypothetical protein	-	-
SCO1726	67.5	73.3	SCO1726	1846425	3.6	-	ATPase	2. P-loop NTPases	2C. Other ATPases
SCO1727	16.8	13.4	SCO1726	-	-	-	hypothetical protein	-	-
SCO1729	153.0	80.3	SCO1729	1848854	8.1	-	hypothetical protein	3. Transferases	3A. GNATs
SCO1730	43.3	25.7	SCO1729	-	-	-	hypothetical protein	-	-

SCO1758	6.3	46.5	SCO1758	1880341	2.1	der	GTP-binding protein EngA	2. P-loop NTPases	2D. GTPases
SCO1795	25.7	111.4	SCO1795	1924436	3.7	-	hypothetical protein	6. Hydrolases	-
SCO1840	3.0	15.7	SCO1840	1969485	27.9	-	ABC transporter ATP-binding protein	2. P-loop NTPases	2B. ABC transporter ATP-binding cassette proteins
SCO1904	26.6	95.0	SCO1905	-	-	-	transcriptional regulator	7. Transcription regulators	-
SCO1905	3084.1	250.0	SCO1905	2040911	26.8	-	hypothetical protein	-	-
SCO1914	64.5	54.3	SCO1914	2049531	5.9	-	hypothetical protein	-	-
SCO1915	21.7	68.6	SCO1914	-	-	-	hypothetical protein	-	-
SCO1940	5.3	28.9	SCO1940	2074165	3.4	-	hypothetical protein	-	-
SCO1987	699.7	79.2	SCO1987	2125725	17.1	-	hypothetical protein	-	-
SCO1988	1025.8	83.5	SCO1987	-	-	-	hypothetical protein	3. Transferases	3A. GNATs
SCO1989	9.6	28.3	SCO1989	2127780	5.5	-	aminopeptidase	6. Hydrolases	-
SCO1991	14.5	21.2	SCO1991	2128663	4.0	hsp15	hypothetical protein	-	-
SCO2076	3.2	16.4	SCO2076	2226968	5.8	ileS	isoleucyl-tRNA synthetase	1. aminoacyl-tRNA synthesis & editing enzymes	-
SCO2235	2.6	7.2	SCO2235	2405927	12.0	yefM	hypothetical protein	-	-
SCO2237	2.3	8.3	SCO2235	-	-	-	lyase	-	-
SCO2248	126.2	31.0	SCO2248	2416296	22.1	-	hypothetical protein	-	-
SCO2249	57.7	97.1	SCO2248	-	-	-	hypothetical protein	-	-
SCO2250	31.0	88.5	SCO2248	-	-	-	hypothetical protein	-	-
SCO2251	42.1	63.8	SCO2248	-	-	-	hypothetical protein	4. Integral membrane proteins	-
SCO2259	6.9	16.9	SCO2259	2430193	7.6	-	multidomain-containing protein family	7. Transcription regulators	-
SCO2264	249.1	40.1	SCO2264	2433284	6.7	-	hypothetical protein	4. Integral membrane proteins	4A. Major facilitator superfamily
SCO2269	13.9	10.1	SCO2269	2438078	3.3	-	hypothetical protein	-	-
SCO2309	24.6	75.3	SCO2309	2479601	4.3	-	transmembrane transport protein	4. Integral membrane proteins	4A. Major facilitator superfamily
SCO2343	12.1	27.8	SCO2343	2511916	4.0	-	acetyltransferase	3. Transferases	3A. GNATs
SCO2372	14.3	38.0	SCO2373	-	-	-	small hydrophobic protein	4. Integral membrane proteins	-
SCO2373	20.1	132.8	SCO2373	2544603	15.4	-	tetracenomycin C efflux protein	4. Integral membrane proteins	4A. Major facilitator superfamily
SCO2379	49.6	45.9	SCO2379	2549405	3.3	-	acetyltransferase	3. Transferases	3A. GNATs
SCO2463	7.6	51.5	SCO2463	2649346	2.3	-	ABC transporter	2. P-loop NTPases	2A. ABC transporters with transmembrane domains
SCO2464	5.7	31.3	SCO2463	-	-	-	ABC transporter	2. P-loop NTPases	2A. ABC transporters with transmembrane domains
SCO2466	65.5	90.6	SCO2466	2654493	9.2	-	hypothetical protein	-	-
SCO2467	35.9	17.5	SCO2466	-	-	-	hypothetical protein	3. Transferases	-
SCO2496	43.4	96.3	SCO2496	2689959	3.6	-	hypothetical protein	-	-
SCO2532	11.1	61.9	SCO2532	2730385	6.3	-	PhoH-like protein	2. P-loop NTPases	2C. Other ATPases
SCO2533	5.3	26.1	SCO2532	-	-	-	metalloprotease	6. Hydrolases	-
SCO2534	5.9	34.9	SCO2532	-	-	-	hypothetical protein	4. Integral membrane proteins	-
SCO2535	5.0	10.1	SCO2532	-	-	-	hypothetical protein	-	-
SCO2586	5.7	4.5	SCO2587	-	-	-	hypothetical protein	-	-
SCO2587	13.0	50.9	SCO2587	2793535	4.7	proB	gamma-glutamyl kinase	-	-
SCO2591	9.5	78.0	SCO2591	2803113	24.3	-	hypothetical protein	-	-
SCO2603	6.9	14.0	SCO2603	2823566	2.4	-	integrase	-	-
SCO2615	3.5	18.7	SCO2615	2841571	6.6	valS	valyl-tRNA synthetase	1. aminoacyl-tRNA synthesis & editing enzymes	-
SCO2625	72.8	105.6	SCO2625	2851869	3.0	eis2	hypothetical protein	3. Transferases	3A. GNATs
SCO2640	11.0	77.4	SCO2640	2870768	10.0	asd1	aspartate-semialdehyde dehydrogenase	5. Oxidoreductases	-
SCO2641	3.1	7.2	SCO2640	-	-	-	resistance protein	-	-
SCO2646	15.0	13.3	SCO2646	2879350	4.7	-	hypothetical protein	4. Integral membrane proteins	-
SCO2675	2.2	2.0	SCO2677	-	-	-	hypothetical protein	-	-
SCO2677	2.7	8.9	SCO2677	2918458	21.4	-	ABC transporter ATP-binding protein	2. P-loop NTPases	2B. ABC transporter ATP-binding cassette proteins
SCO2697	69.1	69.0	SCO2697	2942869	4.2	-	hypothetical protein	-	-
SCO2755	113.9	47.3	SCO2755	3002564	3.7	-	acetyltransferase	3. Transferases	3A. GNATs
SCO2767	4.4	9.6	SCO2768	-	-	-	ATP/GTP-binding protein	2. P-loop NTPases	2C. Other ATPases
SCO2768	8.5	44.9	SCO2768	3019904	3.1	-	hypothetical protein	-	-
SCO2876	18.7	63.9	SCO2876	3130185	2.6	-	acetyltransferase	3. Transferases	3A. GNATs
SCO2877	5.8	20.3	SCO2876	-	-	-	hypothetical protein	4. Integral membrane proteins	-
SCO2878	6.7	17.0	SCO2876	-	-	-	hypothetical protein	-	-
SCO2893	3.2	13.9	SCO2896	-	-	-	hypothetical protein	-	-
SCO2894	72.0	101.1	SCO2896	-	-	-	ABC transporter ATP-binding protein	2. P-loop NTPases	2B. ABC transporter ATP-binding cassette proteins
SCO2895	53.0	140.4	SCO2896	-	-	-	integral membrane transport protein	4. Integral membrane proteins	-
SCO2896	80.6	117.2	SCO2896	3150769	24.2	-	hypothetical protein	4. Integral membrane proteins	-
SCO2964	6.2	16.8	SCO2966	-	-	-	LysR family transcriptional regulator	7. Transcription regulators	-
SCO2966	7.4	39.1	SCO2966	3227524	6.1	smpB	SsrA-binding protein	-	-
SCO2971	2.2	10.6	SCO2971	3231974	22.4	-	hypothetical protein	-	-
SCO2986	3.8	25.0	SCO2986	3257018	15.1	ohrA	hypothetical protein	-	-
SCO3006	31.4	56.4	SCO3006	3280011	6.4	-	acetyltransferase	3. Transferases	3A. GNATs

SCO3105	4.2	16.5	SCO3105	3401963	3.8	-	hypothetical protein	-	-
SCO3106	3.8	19.2	SCO3105	-	-	-	lipoprotein	-	-
SCO3112	14.0	23.2	SCO3112	3413178	2.1	-	hypothetical protein	-	-
SCO3119	3.1	15.3	SCO3119	3421337	2.8	-	hypothetical protein	4. Integral membrane proteins	-
SCO3163	3.3	2.9	SCO3165	-	-	-	hypothetical protein	-	-
SCO3164	30.5	38.6	SCO3165	-	-	-	hypothetical protein	4. Integral membrane proteins	-
SCO3165	24.4	52.4	SCO3165	3467029	2.1	ybaK	hypothetical protein	1. aminoacyl-tRNA synthesis & editing enzymes	-
SCO3217	6.3	24.3	SCO3217	3528931	2.3	cdaR	transcriptional regulator	7. Transcription regulators	-
SCO3277	50.2	48.6	SCO3277	3622310	3.5	-	phosphotransferase	3. Transferases	-
SCO3291	2.2	3.1	SCO3291	3640156	2.8	-	regulatory protein	7. Transcription regulators	-
SCO3294	12.8	22.9	SCO3299	-	-	-	transferase	4. Integral membrane proteins	-
SCO3295	64.3	38.7	SCO3299	-	-	-	oxidoreductase	5. Oxidoreductases	-
SCO3296	66.7	130.7	SCO3299	-	-	-	oxidoreductase	5. Oxidoreductases	-
SCO3299	319.9	248.4	SCO3299	3648633	17.1	rtcB	hypothetical protein	-	-
SCO3303	4.8	38.0	SCO3303	3654092	14.5	lysS	lysyl-tRNA synthetase	1. aminoacyl-tRNA synthesis & editing enzymes	-
SCO3304	3.1	19.5	SCO3304	3654092	14.5	argS	arginyl-tRNA synthetase	1. aminoacyl-tRNA synthesis & editing enzymes	-
SCO3334	2.3	7.0	SCO3334	3691152	21.2	trpS1	tryptophanyl-tRNA synthetase	1. aminoacyl-tRNA synthesis & editing enzymes	-
SCO3360	137.6	119.4	SCO3360	3720023	3.6	-	hypothetical protein	-	-
SCO3366	6.5	43.8	SCO3366	3725489	10.3	-	exporter	4. Integral membrane proteins	4A. Major facilitator superfamily
SCO3385	30.4	50.5	SCO3385	3748904	2.3	-	L-allo-threonine aldolase	-	-
SCO3418	3.6	7.1	SCO3418	3784675	12.6	-	ABC transporter ATP-binding protein	2. P-loop NTPases	2B. ABC transporter ATP-binding cassette proteins
SCO3422	7.0	16.5	SCO3424	-	-	-	hypothetical protein	-	-
SCO3424	5.0	30.2	SCO3424	3789023	11.9	-	regulator	-	-
SCO3538	2.7	7.7	SCO3538	3906828	9.6	-	hypothetical protein	-	-
SCO3581	10.6	62.0	SCO3581	3961095	5.3	-	hypothetical protein	-	-
SCO3631	76.1	122.2	SCO3631	4009355	10.9	-	hypothetical protein	-	-
SCO3633	18.5	30.6	SCO3631	-	-	-	ABC transporter ATP-binding protein	2. P-loop NTPases	2B. ABC transporter ATP-binding cassette proteins
SCO3634	3.7	3.1	SCO3631	-	-	-	ABC transporter membrane-spanning protein	4. Integral membrane proteins	-
SCO3773	4.4	11.4	SCO3773	4147073	7.0	-	LysR family transcriptional regulator	7. Transcription regulators	-
SCO3778	56.0	64.7	SCO3778	4153047	19.3	thrS2	threonyl tRNA synthetase	1. aminoacyl-tRNA synthesis & editing enzymes	-
SCO3795	2.1	4.8	SCO3795	4174573	7.8	aspS	aspartyl-tRNA synthetase	1. aminoacyl-tRNA synthesis & editing enzymes	-
SCO3824	16.7	17.4	SCO3824	4205178	2.1	-	ABC transporter ATP-binding protein	2. P-loop NTPases	2B. ABC transporter ATP-binding cassette proteins
SCO3825	5.4	4.1	SCO3824	-	-	-	ABC-transporter transmembrane protein	4. Integral membrane proteins	-
SCO3910	14.0	70.3	SCO3910	4306379	5.4	-	hypothetical protein	4. Integral membrane proteins	-
SCO3917	40.8	120.4	SCO3917	4313762	6.8	-	hypothetical protein	3. Transferases	3A. GNATs
SCO3940	6.2	37.8	SCO3940	4336407	10.3	-	transmembrane protein	4. Integral membrane proteins	-
SCO3961	4.1	31.6	SCO3961	4361833	8.0	serS	seryl-tRNA synthetase	1. aminoacyl-tRNA synthesis & editing enzymes	-
SCO3978	9.7	26.9	SCO3978	4381974	2.5	-	oxidoreductase	5. Oxidoreductases	-
SCO4007	18.4	47.6	SCO4007	4401519	4.2	-	hypothetical protein	4. Integral membrane proteins	4A. Major facilitator superfamily
SCO4024	9.0	29.7	SCO4024	4421116	20.6	pep	integral membrane efflux protein	4. Integral membrane proteins	4A. Major facilitator superfamily
SCO4092	6.1	45.7	SCO4092	4490647	20.9	hrpA	ATP-dependent helicase	2. P-loop NTPases	2C. Other ATPases
SCO4098	36.9	70.9	SCO4098	4498936	7.6	-	acetyltransferase	3. Transferases	-
SCO4125	11.6	49.2	SCO4125	4537914	6.6	-	acetyltransferase	3. Transferases	3A. GNATs
SCO4177	10.0	43.7	SCO4177	4589456	9.3	-	hypothetical protein	-	-
SCO4178	3.1	2.5	SCO4177	-	-	-	small membrane protein	4. Integral membrane proteins	-
SCO4179	3.2	14.4	SCO4177	-	-	-	hypothetical protein	-	-
SCO4180	3.1	12.0	SCO4177	-	-	nur	iron uptake regulatory protein	7. Transcription regulators	-
SCO4181	2.7	12.8	SCO4177	-	-	-	hypothetical protein	3. Transferases	-
SCO4186	19.8	92.4	SCO4186	4595634	2.1	eis	hypothetical protein	3. Transferases	3A. GNATs
SCO4222	72.5	182.8	SCO4222	4628328	6.4	-	hypothetical protein	-	-
SCO4264	230.0	49.0	SCO4264	4680222	14.7	-	aminoglycoside phosphotransferase	3. Transferases	-
SCO4278	148.7	161.6	SCO4278	4693643	12.6	arfB	peptidyl-tRNA hydrolase domain-containing protein	6. Hydrolases	-
SCO4311	50.7	81.3	SCO4311	4724144	3.9	-	hypothetical protein	3. Transferases	3A. GNATs
SCO4312	2.6	3.1	SCO4312	4724890	6.7	-	hypothetical protein	-	-
SCO4316	21.1	100.4	SCO4316	4729377	20.8	-	ATP/GTP binding protein	2. P-loop NTPases	2C. Other ATPases
SCO4375	9.6	29.1	SCO4375	4789578	6.2	-	MarR family regulatory protein	7. Transcription regulators	-
SCO4376	7.9	13.9	SCO4375	-	-	-	hypothetical protein	-	-
SCO4438	2.0	8.0	SCO4438	4859665	3.9	-	hypothetical protein	4. Integral membrane proteins	4A. Major facilitator superfamily
SCO4448	2.4	4.1	SCO4449	-	-	-	hypothetical protein	-	-
SCO4449	2.9	11.2	SCO4449	4871935	13.1	vgb	hydrolase	-	-
SCO4498	5.6	29.9	SCO4498	4916395	14.3	-	proton transport protein	4. Integral membrane proteins	-
SCO4499	2.2	4.8	SCO4498	-	-	-	TetR family transcriptional regulator	7. Transcription regulators	-
SCO4590	2.5	3.9	SCO4592	-	-	-	hypothetical protein	-	-

SCO4592	7.3	47.0	SCO4592	5015248	4.1	-	hypothetical protein	4. Integral membrane proteins	-
SCO4630	19.2	47.7	SCO4630	5052724	4.3	-	hypothetical protein	-	-
SCO4644	4.9	22.9	SCO4644	5069621	2.1	add	adenosine deaminase	6. Hydrolases	-
SCO4651	14.3	95.1	SCO4651	5075202	7.8	-	lipoprotein	4. Integral membrane proteins	-
SCO4684	2.4	6.7	SCO4684	5113593	3.2	scoF3	cold shock protein	-	-
SCO4763	5.6	13.6	SCO4765	-	-	-	hypothetical protein	-	-
SCO4764	10.2	47.1	SCO4765	-	-	-	dehydrogenase	5. Oxidoreductases	-
SCO4765	95.2	129.2	SCO4765	5177909	11.3	-	hypothetical protein	-	-
SCO4963	12.0	54.6	SCO4963	5398023	4.6	-	ABC transporter ATP-binding protein	2. P-loop NTPases	2B. ABC transporter ATP-binding cassette proteins
SCO4964	5.7	8.1	SCO4963	-	-	-	integral membrane transport protein	4. Integral membrane proteins	-
SCO5024	2.8	12.9	SCO5024	5458027	2.8	-	oxidoreductase	5. Oxidoreductases	-
SCO5025	2.1	2.8	SCO5024	-	-	-	transcriptional regulator	7. Transcription regulators	-
SCO5101	2.3	5.7	SCO5101	5542127	3.9	-	hypothetical protein	-	-
SCO5102	2.2	4.0	SCO5101	-	-	-	mutT-like protein	6. Hydrolases	-
SCO5103	2.2	2.4	SCO5101	-	-	-	regulatory protein	-	-
SCO5189	17.7	57.8	SCO5189	5647154	24.5	-	hypothetical protein	-	-
SCO5190	6330.8	250.0	SCO5189	-	-	wblC	DNA-binding protein	7. Transcription regulators	-
SCO5191	12.4	69.1	SCO5189	-	-	-	hypothetical protein	-	-
SCO5216	3.1	12.7	SCO5216	5675187	5.0	sigR	RNA polymerase sigma factor RpoE	7. Transcription regulators	-
SCO5217	3.2	13.9	SCO5216	-	-	rsrA	anti-sigma factor	-	-
SCO5218	3.6	13.4	SCO5216	-	-	-	hypothetical protein	4. Integral membrane proteins	-
SCO5252	10.7	34.0	SCO5252	5713285	11.3	-	hypothetical protein	5. Oxidoreductases	-
SCO5253	3.4	7.2	SCO5252	-	-	-	hypothetical protein	-	-
SCO5261	2.1	8.7	SCO5261	5719948	5.4	-	malate oxidoreductase	5. Oxidoreductases	-
SCO5333	3.8	7.5	SCO5333	5808298	3.7	-	hypothetical protein	6. Hydrolases	-
SCO5448	4.6	4.7	SCO5451	-	-	-	ABC transporter	2. P-loop NTPases	2A. ABC transporters with transmembrane domains
SCO5449	10.8	26.3	SCO5451	-	-	-	ABC transporter	2. P-loop NTPases	2A. ABC transporters with transmembrane domains
SCO5450	20.9	62.0	SCO5451	-	-	-	ABC transporter	2. P-loop NTPases	2A. ABC transporters with transmembrane domains
SCO5451	34.0	142.7	SCO5451	5937448	20.6	-	ABC transporter	2. P-loop NTPases	2A. ABC transporters with transmembrane domains
SCO5488	2.0	3.6	SCO5488	5973922	2.7	mnmA	tRNA-specific 2-thiouridylase MnmA	-	-
SCO5498	3.4	14.9	SCO5498	5984735	6.0	gatC	aspartyl/glutamyl-tRNA amidotransferase subunit C	1. aminoacyl-tRNA synthesis & editing enzymes	-
SCO5499	2.7	12.8	SCO5498	-	-	gatA	aspartyl/glutamyl-tRNA amidotransferase subunit A	1. aminoacyl-tRNA synthesis & editing enzymes	-
SCO5521	9.1	21.7	SCO5521	6014922	21.0	-	hypothetical protein	-	-
SCO5522	2.8	12.2	SCO5521	-	-	leuB	3-isopropylmalate dehydrogenase	5. Oxidoreductases	-
SCO5552	8.5	20.7	SCO5552	6051803	21.1	ndgR	regulator	7. Transcription regulators	-
SCO5642	4.4	13.3	SCO5642	6141596	7.1	-	hypothetical protein	-	-
SCO5699	6.7	49.3	SCO5699	6209618	9.4	proS	prolyl-tRNA synthetase	1. aminoacyl-tRNA synthesis & editing enzymes	-
SCO5707	6.2	45.0	SCO5707	6219673	14.0	-	hypothetical protein	-	-
SCO5708	5.4	31.5	SCO5707	-	-	rbfA	ribosome-binding factor A	-	-
SCO5709	6.6	59.8	SCO5707	-	-	truB	tRNA pseudouridine synthase B	-	-
SCO5710	5.3	15.1	SCO5707	-	-	-	large Pro/Ala/Gly-rich protein	-	-
SCO5732	2.4	8.8	SCO5733	-	-	-	hypothetical protein	-	-
SCO5733	2.4	7.8	SCO5733	6253404	2.3	-	hypothetical protein	-	-
SCO5791	5.3	26.9	SCO5791	6327659	2.9	miaA	tRNA delta(2)-isopentenylpyrophosphate transferase	2. P-loop NTPases	2C. Other ATPases
SCO5796	18.0	118.7	SCO5796	6334419	15.7	hflX	hypothetical protein	2. P-loop NTPases	2D. GTPases
SCO5813	5.0	19.0	SCO5813	6357011	3.8	-	hypothetical protein	-	-
SCO5814	2.5	4.3	SCO5814	6358875	3.8	-	hypothetical protein	-	-
SCO5848	4.9	25.0	SCO5848	6403548	4.1	agaZ	tagatose 6-phosphate kinase	-	-
SCO5849	3.7	6.3	SCO5848	-	-	agaS	AgaS protein	6. Hydrolases	-
SCO5949	346.9	108.3	SCO5949	6517320	6.0	-	hypothetical protein	-	-
SCO5951	2.3	2.3	SCO5949	-	-	-	transcriptional regulator	7. Transcription regulators	-
SCO5972	132.8	164.3	SCO5972	6543330	8.2	-	3' terminal RNA ribose 2'-O-methyltransferase Hen1	3. Transferases	3B. Methyltransferases
SCO5973	83.3	108.4	SCO5972	-	-	-	phosphatase	6. Hydrolases	-
SCO6075	2.9	10.0	SCO6075	6669904	13.2	cpdA	hypothetical protein	6. Hydrolases	-
SCO6089	406.7	35.3	SCO6089	6687695	17.8	lrm	antibiotic resistance rRNA adenine methyltransferase	3. Transferases	3B. Methyltransferases
SCO6090	20.7	118.3	SCO6090	6688772	17.3	-	antibiotic resistance macrolide glycosyltransferase	3. Transferases	-
SCO6102	2.2	7.9	SCO6102	6702785	13.4	-	nitrite/sulfite reductase	5. Oxidoreductases	-
SCO6149	68.4	231.3	SCO6149	6749958	29.0	rsgA	ribosome-associated GTPase	2. P-loop NTPases	2D. GTPases
SCO6150	95.4	40.3	SCO6149	-	-	-	ADA-like regulatory protein	7. Transcription regulators	-
SCO6151	63.5	60.5	SCO6149	-	-	-	methylated-DNA-protein-cysteine methyltransferase	3. Transferases	3B. Methyltransferases
SCO6222	4.2	20.1	SCO6222	6844371	3.6	-	aminotransferase AlaT	3. Transferases	-
SCO6295	21.8	87.3	SCO6295	6954604	12.8	-	ABC transporter ATP-binding protein	2. P-loop NTPases	2A. ABC transporters with transmembrane domains
SCO6399	33.9	67.6	SCO6399	7065679	20.9	-	hypothetical protein	-	-

SCO6446	15.0	43.4	SCO6446	7130452	2.1	-	hypothetical protein	3. Transferases	3A. GNATs
SCO6463	77.5	39.3	SCO6463	7151629	14.4	-	hypothetical protein	5. Oxidoreductases	-
SCO6464	15.1	44.8	SCO6464	7153295	3.5	cobB2	SIR2 family transcriptional regulator	6. Hydrolases	-
SCO6491	25.7	76.6	SCO6491	7184688	2.5	-	hypothetical protein	-	-
SCO6512	71.6	135.0	SCO6512	7202079	9.0	-	ABC transporter ATP-binding protein	2. P-loop NTPases	2B. ABC transporter ATP-binding cassette proteins
SCO6529	107.7	15.5	SCO6529	7220304	4.0	-	ATP/GTP binding protein	2. P-loop NTPases	2C. Other ATPases
SCO6530	14.8	8.5	SCO6529	-	-	-	hypothetical protein	-	-
SCO6589	86.7	175.2	SCO6589	7300831	17.7	fusB	elongation factor G	2. P-loop NTPases	2D. GTPases
SCO6632	2.2	4.5	SCO6632	7360033	2.9	-	hypothetical protein	-	-
SCO6718	2.7	3.8	SCO6720	-	-	ddah	dimethylarginine dimethylaminohydrolase	6. Hydrolases	-
SCO6719	13.6	75.7	SCO6720	-	-	-	UvrA-like ABC transporter	2. P-loop NTPases	2B. ABC transporter ATP-binding cassette proteins
SCO6720	12.1	93.1	SCO6720	7476881	19.4	-	ABC transporter	2. P-loop NTPases	2B. ABC transporter ATP-binding cassette proteins
SCO6726	2.5	2.6	SCO6726	7480243	2.3	-	endonuclease	6. Hydrolases	-
SCO6805	8.7	8.6	SCO6805	7564447	6.7	-	integral membrane efflux protein	4. Integral membrane proteins	4A. Major facilitator superfamily
SCO6829	2.2	3.7	SCO6829	7601617	3.1	-	oxidoreductase	5. Oxidoreductases	-
SCO6952	33.0	80.1	SCO6952	7716821	3.8	-	hypothetical protein	-	-
SCO6973	18.1	40.7	SCO6973	7741035	2.0	-	hypothetical protein	-	-
SCO7265	51.8	104.5	SCO7265	8075962	11.0	-	hypothetical protein	-	-
SCO7397	84.6	18.9	SCO7397	8210115	2.6	-	hypothetical protein	6. Hydrolases	-
SCO7445	42.5	18.0	SCO7445	8259801	2.2	-	hypothetical protein	3. Transferases	3B. Methyltransferases
SCO7446	3.7	2.7	SCO7445	-	-	-	regulator	-	-
SCO7447	104.5	33.7	SCO7447	8262054	3.1	-	acetyltranferase	3. Transferases	3A. GNATs
SCO7597	2.5	2.5	SCO7600	-	-	-	hypothetical protein	-	-
SCO7600	41.6	21.9	SCO7600	8427408	13.1	alaS2	alanyl tRNA synthetase	1. aminoacyl-tRNA synthesis & editing enzymes	-
SCO7606	19.2	43.8	SCO7606	8433432	2.2	-	amino acid binding protein	-	-
SCO7623	3.5	3.1	SCO7626	-	-	pntA	NAD(P) transhydrogenase subunit alpha	5. Oxidoreductases	-
SCO7624	6.4	17.6	SCO7626	-	-	-	TetR family transcriptional regulator	7. Transcription regulators	-
SCO7626	6.4	52.7	SCO7626	8453839	12.4	-	monooxygenase	5. Oxidoreductases	-
SCO7638	11.5	42.5	SCO7638	8468285	5.5	eno	phosphopyruvate hydratase	-	-
SCO7639	2.0	3.5	SCO7639	8468285	5.5	-	MarR family regulatory protein	7. Transcription regulators	-
SCO7653	101.1	90.3	SCO7653	8480937	6.2	-	hypothetical protein	-	-
SCO7662	6.7	23.6	SCO7662	8488354	5.2	cmlR2	chloramphenicol resistance protein	4. Integral membrane proteins	4A. Major facilitator superfamily
SCO7710	60.9	39.9	SCO7710	8546009	4.1	-	phosphotransferase	3. Transferases	-
SCO7711	3.9	4.8	SCO7710	-	-	-	two component system sensor kinase	4. Integral membrane proteins	-
SCO7731	16.2	47.5	SCO7731	8565519	2.0	-	hypothetical protein	-	-
SCO7806	69.6	96.7	SCO7806	8627142	8.0	-	DNA-binding protein	3. Transferases	3B. Methyltransferases
SCOt18	2.1	4.4	SCOt18	2929568	3.3	-	tRNA-Arg	8. tRNAs	-
SCOt44	2.8	5.7	SCOt44	4490991	19.5	-	tRNA-Glu	8. tRNAs	-
SCOt45	2.9	4.7	SCOt45	4498127	6.8	-	tRNA-Lys	8. tRNAs	-
SCOt55	2.4	5.0	SCOt55	5818803	23.4	-	tRNA-Arg	8. tRNAs	-
SCOt62	2.6	2.4	SCOt62	7300582	26.2	-	tRNA-Leu	8. tRNAs	-
SCOt63	4.0	7.2	SCOt63	7565928	24.9	-	tRNA-Pro	8. tRNAs	-
SCO1087	0.4	4.9	SCO1089	-	-	-	aldolase	-	-
SCO1088	0.4	6.3	SCO1089	-	-	-	oxidoreductase	-	-
SCO1089	0.5	8.1	SCO1089	1149832	2.2	-	hypothetical protein	-	-
SCO1461	0.4	15.8	SCO1461	1559891	2.4	guaB2	inosine 5-monophosphate dehydrogenase	-	-
SCO2015	0.3	13.1	SCO2015	2157903	2.2	-	nucleotidase	-	-
SCO2736	0.3	13.3	SCO2736	2984199	3.7	citA	type II citrate synthase	-	-
SCO3043	0.4	6.7	SCO3043	3330722	4.8	-	hypothetical protein	-	-
SCO3064	0.2	27.0	SCO3064	3356880	2.3	-	peptide transporter	-	-
SCO3607	0.1	48.5	SCO3608	-	-	-	hypothetical protein	-	-
SCO3608	0.2	23.4	SCO3608	3985835	2.2	-	hypothetical protein	-	-
SCO3898	0.3	5.1	SCO3900	-	-	-	hypothetical protein	-	-
SCO3899	0.3	12.8	SCO3900	-	-	-	hypothetical protein	-	-
SCO3900	0.4	7.4	SCO3900	4295576	2.2	-	hypothetical protein	-	-
SCO4240	0.4	9.3	SCO4240	4646508	15.4	msiK	ABC transporter ATP-binding protein	-	-
SCO4251	0.5	5.3	SCO4253	-	-	-	hypothetical protein	-	-
SCO4253	0.5	9.7	SCO4253	4663037	2.4	-	hypothetical protein	-	-
SCO4770	0.3	18.0	SCO4770	5181314	2.9	guaB	inosine 5' monophosphate dehydrogenase	-	-
SCO4914	0.5	5.4	SCO4914	5348224	3.4	-	2-deoxyribose-5-phosphate aldolase	-	-
SCO5240	0.2	32.9	SCO5240	5702241	2.3	wblE	hypothetical protein	-	-
SCO5425	0.4	3.9	SCO5425	5897877	3.0	pta	phosphate acetyltransferase	-	-
SCO5446	0.3	2.9	SCO5446	5927483	2.7	-	hypothetical protein	-	-

SCO5542	0.5	4.2	SCO5544	-	-	cvnC1	hypothetical protein	-	-
SCO5544	0.5	8.8	SCO5544	6044782	3.3	cvnA1	hypothetical protein	-	-
SCO6382	0.4	6.2	SCO6382	7046512	2.6	-	hypothetical protein	-	-
