# Supplementary material for: The WblC/WhiB7 Transcription Factor Controls Intrinsic Resistance to Translation-Targeting Antibiotics by Altering Ribosome Composition
Source: mBio. 2020 Apr 14;11(2):e00625-20. doi: 10.1128/mBio.00625-20 (PMC7157823; doi:10.1128/mBio.00625-20)
Supplement: TABLE S3 [file mBio.00625-20-st003.pdf]

Table S3. Intergenic small RNA and antisense RNA targets of WblC.

Type of RNA	Location: Antisense gene(s) or intergenic region	First gene of operon (UTR/asRNA only)	ChIP-seq peak	
			Summit location	Fold enrichment
intergenic sRNA	SCO0137-SCO0138	-	130881	2.4
intergenic sRNA	SCO4122-SCOr13	-	4530044	4.9
intergenic sRNA	SCO5112-SCO5111	-	5556695	27.7
intergenic sRNA	SCO6373-SCO6372	-	7034272	3.1
asRNA	SCO0236	-	223816	9.2
asRNA	SCO1103	-	1161666	16.9
asRNA	SCO1905	-	2038857	3.9
asRNA	SCO2558	-	2758026	5.1
asRNA	SCO3960, SCO3961	-	4359588	12.2
asRNA	SCO4123, SCO4124	-	4535851	2.5
asRNA	SCO4385, SCO4386, SCO4388	-	4805770	21.6
asRNA	SCO4411	-	4829616	4.6
asRNA	SCO4519	-	4941362	2.1
asRNA	SCO4562	-	4980430	2.3
asRNA	SCO4675, SCO4676	-	5106739	2.3
asRNA	SCO6149	-	6751297	3.3
asRNA	SCO6393	-	7060769	2.1
asRNA	SCO6633	-	7362153	2.3
5'-UTR/asRNA	SCO2698	-	2942869	4.2
5'-UTR/asRNA	SCO3113	-	3413178	2.1
3'-UTR/asRNA	SCO0106	SCO0107	-	-
3'-UTR/asRNA	SCO0164	SCO0165	-	-
3'-UTR/asRNA	SCO0409	SCO0408	-	-
3'-UTR/asRNA	SCO0499	SCO0500	-	-
3'-UTR/asRNA	SCO0524, SCO0525, SCO0526	SCO0527	-	-
3'-UTR/asRNA	SCO0785	SCO0783	-	-
3'-UTR/asRNA	SCO0855, SCO0856	SCO0854	-	-
3'-UTR/asRNA	SCO0905, SCO0908	SCO0909	-	-
3'-UTR/asRNA	SCO1027	SCO1028	-	-
3'-UTR/asRNA	SCO1142	SCO1144	-	-
3'-UTR/asRNA	SCO1149, SCO1150	SCO1151	-	-
3'-UTR/asRNA	SCO1304	SCO1307	-	-
3'-UTR/asRNA	SCO1319	SCO1321	-	-
3'-UTR/asRNA	SCO1342, SCO1343	SCO1340	-	-
3'-UTR/asRNA	SCO1361	SCO1362	-	-
3'-UTR/asRNA	SCO1362	SCO1361	-	-
3'-UTR/asRNA	SCO1411, SCO1412	SCO1413	-	-
3'-UTR/asRNA	SCO1449	SCO1448	-	-
3'-UTR/asRNA	SCO1544	SCO1545	-	-
3'-UTR/asRNA	SCO1622, SCO1623	SCO1624	-	-
3'-UTR/asRNA	SCO1671	SCO1672	-	-
3'-UTR/asRNA	SCO1731	SCO1729	-	-
3'-UTR/asRNA	SCO1796, SCO1797, SCO1798	SCO1795	-	-
3'-UTR/asRNA	SCO1903, SCO1902, SCO1901, SCO1900, SCO1899	SCO1905	-	-
3'-UTR/asRNA	SCO1916, SCO1917	SCO1914	-	-
3'-UTR/asRNA	SCO1938, SCO1939	SCO1940	-	-
3'-UTR/asRNA	SCO1989, SCO1990	SCO1987	-	-
3'-UTR/asRNA	SCO1992	SCO1991	-	-
3'-UTR/asRNA	SCO2238	SCO2235	-	-
3'-UTR/asRNA	SCO2252, SCO2253	SCO2248	-	-
3'-UTR/asRNA	SCO2265	SCO2264	-	-
3'-UTR/asRNA	SCO2308	SCO2309	-	-
3'-UTR/asRNA	SCO2344	SCO2343	-	-
3'-UTR/asRNA	SCO2371	SCO2373	-	-
3'-UTR/asRNA	SCO2465	SCO2463	-	-
3'-UTR/asRNA	SCO2468	SCO2466	-	-
3'-UTR/asRNA	SCO2497	SCO2496	-	-
3'-UTR/asRNA	SCO2536	SCO2532	-	-
3'-UTR/asRNA	SCO2592, SCO2593	SCO2591	-	-
3'-UTR/asRNA	SCO2626, SCO2627	SCO2625	-	-
3'-UTR/asRNA	SCO2695, SCO2696	SCO2697	-	-

3'-UTR/asRNA	SCO2754	SCO2755	-	-
3'-UTR/asRNA	SCO2892	SCO2896	-	-
3'-UTR/asRNA	SCO2970	SCO2971	-	-
3'-UTR/asRNA	SCO2985	SCO2986	-	-
3'-UTR/asRNA	SCO3107	SCO3105	-	-
3'-UTR/asRNA	SCO3117, SCO3118	SCO3119	-	-
3'-UTR/asRNA	SCO3162	SCO3165	-	-
3'-UTR/asRNA	SCO3276	SCO3277	-	-
3'-UTR/asRNA	SCO3297, SCO3298	SCO3299	-	-
3'-UTR/asRNA	SCO3335	SCO3334	-	-
3'-UTR/asRNA	SCO3359	SCO3360	-	-
3'-UTR/asRNA	SCO3423	SCO3424	-	-
3'-UTR/asRNA	SCO3580	SCO3581	-	-
3'-UTR/asRNA	SCO3606	SCO3608	-	-
3'-UTR/asRNA	SCO3632	SCO3631	-	-
3'-UTR/asRNA	SCO3915, SCO3916	SCO3917	-	-
3'-UTR/asRNA	SCO3939	SCO3940	-	-
3'-UTR/asRNA	SCO3977	SCO3978	-	-
3'-UTR/asRNA	SCO4006	SCO4007	-	-
3'-UTR/asRNA	SCO4097, SCOt45	SCO4098	-	-
3'-UTR/asRNA	SCO4098	SCOt45	-	-
3'-UTR/asRNA	SCO4126	SCO4125	-	-
3'-UTR/asRNA	SCO4187	SCO4186	-	-
3'-UTR/asRNA	SCO4223, SCO4224, SCO4225, SCO4226	SCO4222	-	-
3'-UTR/asRNA	SCO4263	SCO4264	-	-
3'-UTR/asRNA	SCO4279, SCO4280	SCO4278	-	-
3'-UTR/asRNA	SCO4309, SCO4310	SCO4311	-	-
3'-UTR/asRNA	SCO4315	SCO4316	-	-
3'-UTR/asRNA	SCO4447	SCO4449	-	-
3'-UTR/asRNA	SCO4591	SCO4592	-	-
3'-UTR/asRNA	SCO4628, SCO4629	SCO4630	-	-
3'-UTR/asRNA	SCO4643	SCO4644	-	-
3'-UTR/asRNA	SCO5239	SCO5240	-	-
3'-UTR/asRNA	SCO5350	SCOt55	-	-
3'-UTR/asRNA	SCO5700	SCO5699	-	-
3'-UTR/asRNA	SCO5797	SCO5796	-	-
3'-UTR/asRNA	SCO5814	SCO5813	-	-
3'-UTR/asRNA	SCO5950	SCO5949	-	-
3'-UTR/asRNA	SCO5974	SCO5972	-	-
3'-UTR/asRNA	SCO6074	SCO6075	-	-
3'-UTR/asRNA	SCO6091	SCO6090	-	-
3'-UTR/asRNA	SCO6152, SCO6153	SCO6149	-	-
3'-UTR/asRNA	SCO6221	SCO6222	-	-
3'-UTR/asRNA	SCO6296, SCO6297	SCO6295	-	-
3'-UTR/asRNA	SCO6383	SCO6382	-	-
3'-UTR/asRNA	SCO6447	SCO6446	-	-
3'-UTR/asRNA	SCO6463	SCO6464	-	-
3'-UTR/asRNA	SCO6464	SCO6463	-	-
3'-UTR/asRNA	SCO6488, SCO6489, SCO6490	SCO6491	-	-
3'-UTR/asRNA	SCO6513	SCO6512	-	-
3'-UTR/asRNA	SCO6590	SCO6589	-	-
3'-UTR/asRNA	SCO6806	SCOt63	-	-
3'-UTR/asRNA	SCO6951	SCO6952	-	-
3'-UTR/asRNA	SCO7263, SCO7264	SCO7265	-	-
3'-UTR/asRNA	SCO7446	SCO7447	-	-
3'-UTR/asRNA	SCO7477	SCO7445	-	-
3'-UTR/asRNA	SCO7598, SCO7599	SCO7600	-	-
3'-UTR/asRNA	SCO7607	SCO7606	-	-
3'-UTR/asRNA	SCO7625	SCO7626	-	-
3'-UTR/asRNA	SCO7652	SCO7653	-	-
3'-UTR/asRNA	SCO7804, SCO7805	SCO7806	-	-
