# Supplementary material for: The WblC/WhiB7 Transcription Factor Controls Intrinsic Resistance to Translation-Targeting Antibiotics by Altering Ribosome Composition
Source: mBio. 2020 Apr 14;11(2):e00625-20. doi: 10.1128/mBio.00625-20 (PMC7157823; doi:10.1128/mBio.00625-20)
Supplement: TABLE S4 [file mBio.00625-20-st004.pdf]

Table S4. Minimum inhibitory concentration (MIC) of various antibiotics.

antibiotics	MIC (µg/ml)*	
	WT	Δ*wblC*
**Lincomycin**	**80**	**10**
**Chloramphenicol**	**40**	**20**
**Fusidic acid**	**0.625**	**0.3125**
**Hygromycin B**	**20**	**10**
**Linezolid**	**1.25**	**0.625**
**Streptomycin**	**2.5**	**1.25**
**Thiostrepton**	**0.4**	**0.2**
Puromycin	640	640
Spectinomycin	160	160

* Median values of three independent experiments are shown.

** Antibiotics showing ≥ 2-fold increase in sensitivity are indicated in bold.
