# Supplementary material for: The WblC/WhiB7 Transcription Factor Controls Intrinsic Resistance to Translation-Targeting Antibiotics by Altering Ribosome Composition
Source: mBio. 2020 Apr 14;11(2):e00625-20. doi: 10.1128/mBio.00625-20 (PMC7157823; doi:10.1128/mBio.00625-20)
Supplement: TEXT S1 [file mBio.00625-20-s0001.pdf]

Text S1. Supplemental methods

## RNA sequencing (RNA-seq) and analysis

Wild-type and Δ*wblC* cells were grown to an $OD_{600}$ of 0.3 to 0.4 and treated with 2 μg/ml tetracycline for 30 min. Control cells were grown in parallel without tetracycline. Then, total RNA was extracted using SDS and hot phenol method and treated with DNase I. Removal of rRNA (Ribo-Zero rRNA Removal Kit, Epicentre) and library construction (TruSeq Stranded Total RNA Sample Prep Kit, Illumina) were performed according to the manufacturer's instructions. Sequencing was performed using an Illumina HiSeq 4000 by 101 bp paired-end reading. Quality of reads was assessed (FastQC v0.11.4; www.bioinformatics.babraham.ac.uk/projects/fastqc), adapter sequences were trimmed (Trimmomatic v0.36) (1), and the processed reads were mapped to the *S. coelicolor* A3(2) reference genome NC_003888.3 (Bowtie v2.3.2) (2). Raw counts for various features were obtained using featureCounts from the subread package (v1.5.3) (3), normalized or analysed for differential expression (DESeq2 package of Bioconductor v3.5) (4), and visualized (Integrative Genomics Viewer v2.3.72) (5). Principal component analysis of DESeq2-normalized values was performed on ClustVis using default parameters (6).

## Chromatin immunoprecipitation-sequencing (ChIP-seq) and analysis

ChIP-seq was performed as described previously (7) with minor modifications. Cultures at an $OD_{600}$ of 0.3 to 0.4 were treated with 2 μg/ml tetracycline for an hour and then subjected to formaldehyde crosslinking followed by glycine quenching. Harvested and washed cells were sonicated with Q500 Sonicator (Qsonica) equipped with a 3.2 mm micro-tip for 8 times at 20% maximum power with 20 sec pulses. After cell debris was removed by centrifugation, cell lysate was pre-cleared with 20 μl of protein A/G bead slurry (Santa Cruz). Prior to immunoprecipitation, aliquots of the equal $A_{260}$ unit of lysates were kept as input DNA controls. 10 μl of polyclonal anti-WblC antibodies were added to the pre-cleared lysates and incubated at 4°C for 1 h with gentle mixing by rotation. Subsequently, 50 μl of protein A/G beads were added and incubated overnight at 4°C. After washing the beads, protein-DNA complexes were eluted from beads and then the eluates were treated with proteinase K and RNase A. The lysates for the input DNA control were also treated with proteinase K and RNase A. After a reverse-crosslinking overnight in a high concentration of NaCl, DNA was purified by phenol-chloroform extraction. Three biologically independent samples were pooled separately for immunoprecipitated DNA and input DNA. DNA libraries were prepared using KAPA DNA Library

Preparation Kit (Roche) and sequenced using an Illumina HiSeq 4000 by 101 bp paired-end reading. Quality check, adapter trimming, and mapping of the reads were performed as described for the RNA-seq. Duplicated reads were identified and removed (MarkDuplicates of Picard v1.133; broadinstitute.github.io/picard). We used input genomic DNA without immunoprecipitation as a control, and the significantly enriched peak regions in ChIP samples ($q$-value<0.01) compared to input genomic DNA were determined (MACS v2.1.1) (8). Please note that the specificity of the polyclonal anti-WblC antibodies was tested by western blots using protein extracts prepared from tetracycline-treated wild-type and Δ*wblC* mutant cells and was confirmed that cross-reactive bands were undetectable in the Δ*wblC* mutant.

**Chromatin immunoprecipitation-quantitative PCR (ChIP-qPCR)**

ChIP DNA sample preparation was performed as described above in the ChIP-seq section, with several modifications. 20 μl of protein A/G beads pre-incubated with BSA and either 5 μl of anti-WblC or 2 μl of anti-HrdB polyclonal rabbit antibodies were added to the cell lysates. The mixture was incubated overnight at 4°C with rotatory mixing. Protein-DNA complex elution, proteinase K treatment, RNase A treatment, reverse-crosslinking, and DNA purification were performed as described in the ChIP-seq procedure. qPCR was performed using TOPreal qPCR 2× PreMIX (Enzynomics) for ChIP samples and input DNA samples to obtain percent input values. Relative % input represents [% input in wild-type/% input in Δ*wblC*] in the presence of erythromycin.

**Motif prediction from WblC-activated promoters**

MEME from the MEME Suite (v5.0.4) was used for motif prediction from WblC-activated promoter sequences (9). A 4-order Markov model generated from the *S. coelicolor* A3(2) genome sequence was used as a background model for strand-specific search. After approximate transcription start sites (TSSs) were determined from the 5' ends of differentially expressed RNA in RNA-seq data, an initial motif search for the WblC binding site and the –35 element of the given genes was performed from –65 to –25 relative to the approximate TSSs. The –10 element motifs of each gene were analysed separately from –35 to –5 relative to the approximate TSSs. Then, two sets of the retrieved data from the initial motif searches were cross checked to determine whether two sets of motifs were overlapped each other with $p$-value<0.05 for both motifs. The data were further curated by checking whether the spacer DNA lengths between –35 and –10 elements ranged from 16 to 19 bp. The resulting motifs were combined and visualized as sequence logos (WebLogo v2.8.2) (10).

**Functional analysis and categorization of WblC regulon genes**

For all *S. coelicolor* chromosomal protein-coding genes, Gene Ontology (released 2. 2. 2019.) (11) annotations by PANTHER Classification System (v14.1) (12), InterPro entry (v73.0) annotations (13), and EggNOG (v4.5.1) (14) annotations using EggNOG-mapper (15) were utilized for functional analysis of WblC regulon genes. Enrichment of each functional classes (GO term, InterPro entry, or EggNOG) in either WblC-activated or WblC-repressed genes compared to all chromosomal protein-coding genes were assessed by Fisher’s exact test, for all annotated classes within the genome. Functional classes were determined as significantly enriched if the FDR-adjusted *p*-value was below 0.05. WblC-activated genes were hierarchically clustered according to GO terms and InterPro annotations via ClustVis (6). The resulting clusters were further amended to visualize functional categories of WblC-activated genes.

**Construction of mutant and complemented strains**

*Streptomyces coelicolor* mutant strains disrupting *hrpA*, *hflX*, *arfB*, or *helY* genes were constructed by PCR-targeted mutagenesis (16). In brief, disruption cassettes targeting each gene were generated using PCR primers listed in Table S1. The amplified PCR products were recombined into *S. coelicolor* cosmids D17 (for *hrpA*), 4H2 (for *hflX*), D86A (for *arfB*), or I46 (for *helY*) via the λ-Red mediated system of BW25113/pIJ790. The recombined cosmids were conjugated into M145 cells using *E. coli* ET12567/pUZ8002. Apramycin-resistant and kanamycin-sensitive double-crossover mutants were selected, and the replacement of each gene by disruption cassette was confirmed by PCR. For a SCO2532 deletion mutant, flanking sequences of SCO2532 were amplified by PCR and cloned into the pKC1139 vector (17). The vector carrying the SCO2532 flanking sequences was conjugated into M145 cells using *E. coli* ET12567/pUZ8002. Apramycin-resistant single-crossover exconjugants were selected at 37°C, confirmed by PCR, and sub-cultured to obtain the double-crossover. An apramycin-sensitive double-crossover mutant was selected and confirmed by PCR. The *hrpA*, *hflX*, and SCO2532 mutant strains were complemented using pSET162, an integrative vector with thiostrepton resistance marker that has been previously described (18). pSET162 plasmids carrying *hrpA*, *hflX*, or SCO2532 genes were conjugated into corresponding mutant strains via *E. coli* strain ET12567/pUZ8002. Thiostrepton-resistant exconjugants were selected and vector integration was confirmed by PCR.

**References**

1. Bolger AM, Lohse M, Usadel B. 2014. Trimmomatic: a flexible trimmer for Illumina sequence data. Bioinformatics 30:2114-20.
2. Langmead B, Salzberg SL. 2012. Fast gapped-read alignment with Bowtie 2. Nat Methods 9:357-9.
3. Liao Y, Smyth GK, Shi W. 2014. featureCounts: an efficient general purpose program for assigning sequence reads to genomic features. Bioinformatics 30:923-30.
4. Love MI, Huber W, Anders S. 2014. Moderated estimation of fold change and dispersion for RNA-seq data with DESeq2. Genome Biol 15:550.
5. Thorvaldsdottir H, Robinson JT, Mesirov JP. 2013. Integrative Genomics Viewer (IGV): high-performance genomics data visualization and exploration. Brief Bioinform 14:178-92.
6. Metsalu T, Vilo J. 2015. ClustVis: a web tool for visualizing clustering of multivariate data using Principal Component Analysis and heatmap. Nucleic Acids Res 43:W566-70.
7. Yoo JS, Oh GS, Ryoo S, Roe JH. 2016. Induction of a stable sigma factor SigR by translation-inhibiting antibiotics confers resistance to antibiotics. Sci Rep 6:28628.
8. Zhang Y, Liu T, Meyer CA, Eeckhoute J, Johnson DS, Bernstein BE, Nusbaum C, Myers RM, Brown M, Li W, Liu XS. 2008. Model-based analysis of ChIP-Seq (MACS). Genome Biol 9:R137.
9. Bailey TL, Boden M, Buske FA, Frith M, Grant CE, Clementi L, Ren J, Li WW, Noble WS. 2009. MEME SUITE: tools for motif discovery and searching. Nucleic Acids Res 37:W202-8.
10. Crooks GE, Hon G, Chandonia JM, Brenner SE. 2004. WebLogo: a sequence logo generator. Genome Res 14:1188-90.
11. The Gene Ontology Consortium. 2019. The Gene Ontology Resource: 20 years and still GOing strong. Nucleic Acids Res 47:D330-D338.
12. Mi H, Muruganujan A, Ebert D, Huang X, Thomas PD. 2019. PANTHER version 14: more genomes, a new PANTHER GO-slim and improvements in enrichment analysis tools. Nucleic Acids Res 47:D419-D426.
13. Mitchell AL, Attwood TK, Babbitt PC, Blum M, Bork P, Bridge A, Brown SD, Chang HY, El-Gebali S, Fraser MI, Gough J, Haft DR, Huang H, Letunic I, Lopez R, Luciani A, Madeira F, Marchler-Bauer A, Mi H, Natale DA, Necci M, Nuka G, Orengo C, Pandurangan AP, Paysan-Lafosse T, Pesseat S, Potter SC, Qureshi MA, Rawlings ND, Redaschi N, Richardson LJ, Rivoire C, Salazar GA, Sangrador-Vegas A, Sigrist CJA, Sillitoe I, Sutton GG, Thanki N, Thomas PD, Tosatto SCE, Yong SY, Finn RD. 2019. InterPro in 2019: improving coverage, classification and access to protein sequence annotations. Nucleic Acids Res 47:D351-D360.
14. Huerta-Cepas J, Szklarczyk D, Forslund K, Cook H, Heller D, Walter MC, Rattei T, Mende DR, Sunagawa S, Kuhn M, Jensen LJ, von Mering C, Bork P. 2016. eggNOG

4.5: a hierarchical orthology framework with improved functional annotations for eukaryotic, prokaryotic and viral sequences. Nucleic Acids Res 44:D286-93.

15. Huerta-Cepas J, Forslund K, Coelho LP, Szklarczyk D, Jensen LJ, von Mering C, Bork P. 2017. Fast Genome-Wide Functional Annotation through Orthology Assignment by eggNOG-Mapper. Mol Biol Evol 34:2115-2122.
16. Fowler-Goldsworthy K, Gust B, Mouz S, Chandra G, Findlay KC, Chater KF. 2011. The actinobacteria-specific gene *wblA* controls major developmental transitions in *Streptomyces coelicolor* A3(2). Microbiology 157:1312-28.
17. Bierman M, Logan R, O'Brien K, Seno ET, Rao RN, Schoner BE. 1992. Plasmid cloning vectors for the conjugal transfer of DNA from Escherichia coli to Streptomyces spp. Gene 116:43-9.
18. Kim IK, Lee CJ, Kim MK, Kim JM, Kim JH, Yim HS, Cha SS, Kang SO. 2006. Crystal structure of the DNA-binding domain of BldD, a central regulator of aerial mycelium formation in *Streptomyces coelicolor* A3(2). Mol Microbiol 60:1179-93.
